# Smoking-Associated Exposure of Human Primary Bronchial Epithelial Cells to Aldehydes: Impact on Molecular Mechanisms Controlling Mitochondrial Content and Function

**DOI:** 10.3390/cells11213481

**Published:** 2022-11-03

**Authors:** Christy B. M. Tulen, Evert Duistermaat, Johannes W. J. M. Cremers, Walther N. M. Klerx, Paul H. B. Fokkens, Naömi Weibolt, Nico Kloosterboer, Mieke A. Dentener, Eric R. Gremmer, Phyllis J. J. Jessen, Evi J. C. Koene, Lou Maas, Antoon Opperhuizen, Frederik-Jan van Schooten, Yvonne C. M. Staal, Alexander H. V. Remels

**Affiliations:** 1 School of Nutrition and Translational Research in Metabolism (NUTRIM), Department of Pharmacology and Toxicology, Maastricht University Medical Center+, 6200 MD Maastricht, The Netherlands; 2 National Institute for Public Health and the Environment (RIVM), 3721 MA Bilthoven, The Netherlands; 3 Department of Pediatrics, Maastricht University Medical Center+, 6229 HX Maastricht, The Netherlands; 4 Primary Lung Culture (PLUC) Facility, Maastricht University Medical Center+, 6200 MD Maastricht, The Netherlands; 5 School of Nutrition and Translational Research in Metabolism (NUTRIM), Department of Respiratory Medicine, Maastricht University Medical Center+, 6200 MD Maastricht, The Netherlands; 6 Office of Risk Assessment and Research, Netherlands Food and Consumer Product Safety Authority (NVWA), 3511 GG Utrecht, The Netherlands

**Keywords:** acetaldehyde, acrolein, formaldehyde, cigarette smoke, primary bronchial epithelial cells, mitochondria, inhalation toxicology

## Abstract

Chronic obstructive pulmonary disease (COPD) is a devastating lung disease primarily caused by exposure to cigarette smoke (CS). During the pyrolysis and combustion of tobacco, reactive aldehydes such as acetaldehyde, acrolein, and formaldehyde are formed, which are known to be involved in respiratory toxicity. Although CS-induced mitochondrial dysfunction has been implicated in the pathophysiology of COPD, the role of aldehydes therein is incompletely understood. To investigate this, we used a physiologically relevant in vitro exposure model of differentiated human primary bronchial epithelial cells (PBEC) exposed to CS (one cigarette) or a mixture of acetaldehyde, acrolein, and formaldehyde (at relevant concentrations of one cigarette) or air, in a continuous flow system using a puff-like exposure protocol. Exposure of PBEC to CS resulted in elevated IL-8 cytokine and mRNA levels, increased abundance of constituents associated with autophagy, decreased protein levels of molecules associated with the mitophagy machinery, and alterations in the abundance of regulators of mitochondrial biogenesis. Furthermore, decreased transcript levels of basal epithelial cell marker *KRT5* were reported after CS exposure. Only parts of these changes were replicated in PBEC upon exposure to a combination of acetaldehyde, acrolein, and formaldehyde. More specifically, aldehydes decreased *MAP1LC3A* mRNA (autophagy) and BNIP3 protein (mitophagy) and increased ESRRA protein (mitochondrial biogenesis). These data suggest that other compounds in addition to aldehydes in CS contribute to CS-induced dysregulation of constituents controlling mitochondrial content and function in airway epithelial cells.

## 1. Introduction

Inhalation of cigarette smoke (CS) is responsible for 6.5 million deaths annually as a result of tobacco-related diseases including chronic obstructive pulmonary disease (COPD) [1,2]. CS contains over 6000 chemicals [3]. One class of chemicals well-known to be generated during the pyrolysis and combustion of tobacco are aldehydes. These include the short-chain aldehydes acetaldehyde, acrolein, and formaldehyde, which have similar mechanisms of formation, molecular structures, and chemical properties [4,5,6]. The airways of smokers are exposed to these aldehydes in peak concentrations [7]. Smoking-associated exposure to these aldehydes are thought to induce cellular mechanisms underlying respiratory toxicity, which have been studied particularly in response to acrolein [8,9,10]. Taking into consideration their chemical characteristics and detrimental impact on health, these three short-chain aldehydes are considered representative for the chemical class of aldehydes present in CS of all brands and human smoking regimes [4,5,6,11] and have been shortlisted for regulation in CS by the World Health Organization Study Group on Tobacco Product Regulation [12,13].

Inhaled toxicants, such as compounds that are abundantly present in CS, primarily reach the epithelial cells of the respiratory tract [14], which include a variety of cell types such as ciliated, club, and goblet cells [15,16]. The mitochondria present in these distinct cell types differ in number and intracellular organization, and are essential for proper function of these cell types (e.g., ciliary function and mucus production) [17,18]. Moreover, mitochondria play a key role in the regulation of inflammation, oxidative stress, and cell death [17], all of which are processes known to be involved in the pathogenesis of COPD [19]. As a relevant example, mitochondrial dysfunction induces production of pro-inflammatory cytokines in alveolar epithelial cells [20]. Moreover, based on recent evidence, a mechanistic link has been suggested between mitochondrial-derived reactive oxygen species and the mediation of mitochondrial dysfunction-induced inflammatory processes, which can contribute to the pathogenesis of inflammation-related diseases such as COPD [21].

Mitochondrial homeostasis is regulated by several processes which together coordinate adequate quality control of mitochondria. These processes include mitophagy, mitochondrial biogenesis, and mitochondrial fission and fusion [17]. Mitophagy is responsible for the breakdown of damaged or defective mitochondria via autophagy. The degradation of mitochondria is triggered by activation of mitochondrial receptors (i.e., receptor-mediated mitophagy) or a loss of membrane potential resulting in accumulation or activation of specific proteins (i.e., ubiquitin-mediated mitophagy) [22]. To replenish damaged or destroyed mitochondria, the generation of new mitochondria (i.e., mitochondrial biogenesis) is facilitated via the peroxisome proliferator-activated receptor gamma, coactivator 1 (PPARGC1) signaling network accompanied by PPARGC1 alpha coactivators [23,24]. The dynamic processes of fission and fusion are essential in facilitating these mitochondrial quality-control processes [25].

Emerging evidence suggests a role for CS-induced mitochondrial dysfunction in the airway pathogenesis of COPD [18,26,27]. Indeed, abnormal mitochondrial morphology has been observed in airway epithelial cells from COPD patients and in several experimental smoke-exposure models in vivo and in vitro [28,29,30,31,32,33,34,35,36,37]. Moreover, the literature describes an upregulation of key regulators controlling the mitophagy machinery and constituents associated with autophagy in various in vitro smoke-exposure models of human bronchial epithelial cells [31,33,37,38,39,40,41,42,43,44,45,46,47]. Importantly, Cloonan et al. also demonstrated that blocking of CS-induced mitochondrial dysfunction in mice prevented the development of COPD-like features such as bronchitis and emphysema [29].

The exact chemical constituents of CS responsible for these mitochondrial abnormalities are unknown. Interestingly, Morita et al. previously showed an association between the presence of an inactive allele of aldehyde dehydrogenase (an enzyme that detoxifies aldehydes) and increased incidence of smoking-related chronic airway obstruction in a Japanese population [48]. Moreover, it has been shown that in vivo acrolein exposure resulted in impaired lung function and structure [49].

Studies assessing the impact of aldehydes on mitochondrial function in cells of the airways are scarce. The available studies focus primarily on the impact of individual aldehydes on mitochondrial metabolism, in particular the most reactive aldehyde acrolein. As CS consists of a complex combination of aldehydes, varying in concentration due to tobacco brand and smoking regime [11], these abovementioned individual aldehyde-exposure studies are not representative of the exposure of airways of smokers to aldehydes. Moreover, airborne exposure methods (such as we deployed) have not commonly been used in studies regarding aldehydes or CS, due to the complexity of the models.

Therefore, the aim of this study was to investigate the impact of a mixture of three aldehydes (representative for CS) on a comprehensive panel of constituents involved in molecular mechanisms controlling mitochondrial content and mitochondrial quality-control (mitochondrial biogenesis vs mitophagy). We used a physiologically relevant in vitro model of the human bronchial epithelium. To this end, fully differentiated human primary bronchial epithelial cells (PBEC) from non-COPD subjects (n = 4) were exposed to CS (1 cigarette), or a mixture of acetaldehyde, acrolein, and formaldehyde (at concentrations equivalent to one cigarette), or air (control), in a continuous flow system using a puff-like exposure protocol. After recovery for 6 h or 24 h, suitable timepoints to detect CS-induced changes at transcript and protein levels, respectively [50,51], we assessed cytotoxicity, inflammatory protein secretion, and ciliary beating, as well as protein and transcript levels of key constituents involved in mitochondrial metabolic pathways and quality control. With regard to these timepoints, we and others have previously shown that 6 h and 24 h after CS exposure are adequate timepoints to detect changes in the regulatory pathways controlling mitochondrial content and function (mitochondrial biogenesis vs mitophagy) respectively, at the mRNA and protein levels [50,51]. We hypothesized that acute exposure to a mixture of aldehydes, representative for CS, disrupts the molecular regulation of mitochondrial content and mitochondrial function.

## 2. Materials and Methods

### 2.1. PBEC Isolation

Lung tissue used for the isolation of PBEC was obtained from the Maastricht Pathology Tissue Collection (MPTC). The scientific board of the MPTC (MPTC 2010-019) and the local Medical Ethic Committee (METC 2017-0087; date: 19 October 2017) provided approval for use of lung tissue for research purposes. In line with the Human Tissue and Medical Research Code of Conduct for Responsible Use, the available lung tissue was coded and handled anonymously with respect to patient data, tissue collection, storage, and further use, within the framework of patient care at Maastricht University Medical Center+ (MUMC+). Isolation, culture, and characterization of cells were performed by the Primary Lung Culture (PLUC) facility at the MUMC+ as previously described [52,53]. PBEC were isolated from resected lung tissue of 4 patients without known history of chronic lung disease, who underwent surgery for solitary pulmonary nodules. Characteristics of the subjects are described in Table 1.

### 2.2. PBEC Proliferation and Differentiation

PBEC of four donors without known history of COPD (5 × 10^5^ cells, passage 1) were thawed, seeded, and expanded in T75-flasks (passage 2; Corning Costar, Corning, NY, USA) on pre-coated growth areas (coating: BSA, human fibronectin, PureCol Type I collagen solution) in Pneumacult^TM^-Ex medium including Pneumacult^TM^-Ex basal medium, Pneumacult^TM^-Ex 50× supplement, hydrocortisone stock solution (96 µg/mL) (all StemCell Technologies, GmbH, Cologne, Germany) and 1% penicillin/streptomycin (10.000 U/mL/10.000 μg/mL) (Gibco, Billings, MT, USA) following the manufacturer’s protocol for Pneumacult^TM^-Ex medium (#5008; StemCell Technologies). PBEC were washed every other day with Hank’s Balanced Salt Solution (HBSS; no calcium, no magnesium, no phenol red) (Gibco) followed by refreshment of the Pneumacult^TM^-Ex medium. Upon reaching 80–90% confluency (approximately 5–7 days of proliferation), PBEC were seeded at a density of 40.000 cells (passage 3) on pre-coated 1.12 cm^2^ polycarbonate membrane cell-culture inserts (Corning) using the Pneumacult^TM^-Ex medium. Upon reaching 90% confluency (approximately 5–7 days of proliferation) on the inserts, PBEC were airlifted by removing apical medium. Differentiation at the air–liquid interface (ALI) was maintained for 28–32 days by replacing basolateral medium with Pneumacult^TM^-ALI maintenance medium containing Pneumacult^TM^-ALI basal medium, Pneumacult^TM^-ALI 10× supplement, Pneumacult^TM^-ALI maintenance 100× supplement, heparin solution (0.2%), hydrocortisone stock solution (96 µg/mL) (all StemCell Technologies) and 1% penicillin/streptomycin (10.000 U/mL/10.000 μg/mL) (Gibco) following the manufacturer’s protocol for Pneumacult^TM^-ALI maintenance medium (#5001; StemCell Technologies). PBEC were washed every other day with HBSS, both apically and basolaterally, and the basolateral Pneumacult^TM^-ALI maintenance medium was subsequently changed. PBEC were differentiated for up to 28–32 days in an incubator at 37 °C with 5% CO_2_ in humidified air, until exposure.

### 2.3. Validation and Characterization of Differentiation of PBEC at ALI

To validate and characterize our model of differentiation of PBEC at the ALI using the above-mentioned culturing protocol, we analyzed mRNA expression and immunohistochemistry staining of cell-type-specific markers associated with basal and luminal cells from two representative donors. Undifferentiated PBEC (day 0; moment of airlift) and PBEC during differentiation (days 14, 21, and 28 post-airlift) were harvested in TRIzol^TM^ reagent or fixed in 4% paraformaldehyde in PBS (all from Thermo Fisher Scientific, Waltham, MA, USA) to evaluate the abundance of basal and luminal characterization markers reflecting differentiation status by gene-expression analysis (see Section 2.7: RNA isolation and real-time quantitative PCR) and immunohistochemistry (see Section 2.9: Fixation for paraffin-embedded section and immunohistochemistry staining).

### 2.4. Monitoring PBEC Differentiation and Monolayer Integrity

Several measurements were taken during the differentiation of the PBEC to validate the development of a basal monolayer into a pseudostratified epithelium, for all four donors. First, monolayer integrity was monitored during differentiation of all PBEC cultures from all donors before exposure to CS and aldehydes. The trans-epithelial electrical resistance (TEER) was assessed at day 0 (day of airlift) in apical and basolateral Pneumacult^TM^-Ex medium, and post-airlift at days 7, 14, 21, and 28, as well as 24 h before exposure (this timepoint was in some cases equivalent to day 28 post-airlift) in apical HBSS or Pneumacult^TM^-ALI maintenance medium and basolateral Pneumacult^TM^-ALI maintenance medium. An epithelial volt–ohm meter (World Precision Instruments, Sarasota, FL, USA) was employed to analyze the TEER of the PBEC cultures as indication of an intact monolayer of the pseudostratified epithelium. TEER (Ω·cm^2^) was corrected for the surface of the 12-well inserts and the background measurements of the medium used. Then, cell-layer permeability was analyzed using fluorescein isothiocyanate-dextran (FITC-dextran) immediately after analysis of the bead motion in a flow induced by ciliary beating. FITC-dextran solution (Sigma-Aldrich, Saint Louis, MO, USA; 1 mg/mL) was prepared in Pneumacult^TM^-ALI maintenance medium. Subsequently 0.5 mL FITC-dextran was added to the apical side of the inserts. After incubation in the dark for 2 h in the incubator at 37 °C and 5% CO_2_ in humidified air, the inserts were transported to a new 12-well plate to prevent spillage and to guarantee similar incubation times among the inserts. Technical triplicates of 100 µL of the basolateral medium for each insert were analyzed by fluorescence measurement (Molecular Devices, San Jose, CA, Spectramax M2) (485/535 nm; intensity 7000) in a 96-well plate (black; Corning Costar). Fluorescence was corrected for background of Pneumacult^TM^-ALI maintenance medium and expressed relative to 2% Triton-X-100 (i.e., cell lysis resulting in complete permeability of the membrane).

### 2.5. Dosimetry and Exposure Regime

As shown in Supplementary Figure S1, an experimental puff-like exposure system was designed to expose differentiated PBEC cultures directly to CS or to a mixture of aldehydes (at concentrations equivalent to 1 cigarette) via the air, using two Vitrocell^®^ 12/3 CF stainless steel modules for 12-well sized inserts (Vitrocell, Waldkirch, Germany). Exposure to CS or aldehydes was conducted according to the Health Canada Intense (HCI) exposure regime. We opted for Marlboro Red as a choice of tobacco product as Marlboro Red is one of the leading commercially-available brands worldwide. Moreover, it has been shown that smoking of Marlboro Red resulted in exposure to concentrations of this study’s three target aldehydes in higher ranges than 11 analyzed commercial brands (11). Importantly, the concentrations are comparable in Marlboro Red and 3R4F (the most frequently used reference cigarette in the literature). Specifically, 8 puffs were taken by the smoking machine (VC1, Vitrocell, Waldkirch, Germany) from 1 cigarette (Marlboro Red; taped) or the gaseous mixture of aldehydes in the buffer tank following the HCI Bell regime (2 puffs/min, 55 mL puff volume, 2 s puff duration, 4 s puff exhaust time; taped). The generated streams of CS or aldehydes were distributed (and diluted) in the exposure manifold by a mass-flow-controlled stream of 500 mL/min compressed dry air. Subsequently, streams of 5 mL/min per insert were extracted from the exposure manifold. These streams were individually humidified by passage through Nafion humidifiers in a water bath at 33.8 °C. After humidification, the streams were delivered to the cells. As control, the PBEC cultures were exposed to clean compressed air with a similar flow rate and similar humidification within the control module.

To match the target concentrations of aldehydes present in the mixture to the concentrations present in CS of one Marlboro Red cigarette, we analyzed the total concentrations of three aldehydes, i.e., acetaldehyde, acrolein, and formaldehyde, in a Marlboro Red cigarette. Marlboro Red cigarettes were taped following the World Health Organization SOP1 standard operating procedure for intense smoking of cigarettes [54] and subsequently ‘smoked’ following the above-described HCI regime. Directly prior to the measurement of aldehydes in the CS, the exposure system was primed by smoking 3 cigarettes. After priming, three single cigarettes were consecutively smoked to analyze the aldehydes present in the smoke. The stream of smoke from one cigarette was trapped using a Cambridge filter and Carboxen adsorbent cartridge (external filter) placed next to the smoking machine. Next to using the primary trapping cartridge, an additional cartridge was included to measure possible flow through. The conditions were 1 L/min continuous sample flow, using an exhaust-flow smoking machine complemented with clean compressed air. Extraction and analysis of the trapped aldehydes followed the World Health Organization TobLabNet SOP8 for determination of aldehydes in mainstream CS under International Standardization Organization (ISO) intense smoking conditions, with the exception of 1 min extraction instead of 30 min [55]. Flow through was measured for acetaldehyde and formaldehyde in the CS (~10–15%), and the mean concentrations of all three aldehydes including flow through (mg/m^3^) measured in the CS of 1 Marlboro Red cigarette were as expected compared to the literature (Supplementary Table S1) [11]. Based on these concentrations, the target concentrations of the mixture of aldehydes were set.

The mixture of 3 aldehydes was generated by evaporating syringe-pump-controlled flows of aldehydes in streams of mass-flow-controlled compressed dry air, using two Dimroth evaporators (one for formaldehyde, the other for acrolein and acetaldehyde). After evaporation, the generated formaldehyde and acrolein/acetaldehyde streams were mixed in a buffer chamber. To reach the target concentrations of each aldehyde, the target settings of the syringe pumps were calculated using the density and purity of each aldehyde, and the total flow of the test atmosphere. For formaldehyde, a flow of 2.55 µL/min 37 wt% formaldehyde solution (Sigma Aldrich, Saint Louis, MO, USA) with a density of 1.09 mg/µL was evaporated in a stream of 5 L/min of compressed dry air, to obtain a calculated concentration of 102.8 mg/m^3^ (83 ppm). A flow of 1.64 µL/min of pure acrolein (Sigma Aldrich) with a density of 0.839 mg/µL was evaporated in a stream of 5 L/min compressed air, together with 27.99 µL/min of pure acetaldehyde (Sigma Aldrich) with a density of 0.76 mg/µL. The calculated concentrations of acrolein and acetaldehyde were 137.5 mg/m^3^ (59 ppm) and 2126 mg/m^3^ (1162 ppm), respectively.

A total carbon analyzer (TCA, Ratfisch, Poing, Germany) was employed to monitor the concentrations of aldehydes during every exposure. The response of the TCA was used as an indicator of the presence of each of the aldehydes in the gaseous mixture (located before the smoking machine); for an example of the TCA measurement during exposure, see Supplementary Figure S2. Together with the nominal calculated concentrations, this confirmed the exposure of the cells to each of the aldehydes.

### 2.6. PBEC Exposure to CS or Mixture of Aldehydes 

PBEC of four donors without known history of COPD were differentiated for up to 29–32 days until exposure. 24 h prior to treatment, fully differentiated PBEC were apically and basolaterally washed with HBSS, followed by basolateral replacement of the Pneumacult^TM^-ALI maintenance medium. PBEC were transported in a portable incubator at 37 °C to the laboratory for exposure.

After priming the system, as explained above, the PBEC were placed into Vitrocell^®^ 12/3 CF stainless steel modules (exposure or control module) which were preheated to 37 °C and filled with fresh Pneumacult^TM^-ALI maintenance medium. PBEC were exposed to 8 puffs of CS, aldehyde mixture, or compressed dry air following the HCI smoking regime described in Section 2.5. Immediately after exposure to CS or the mixture of aldehydes, PBEC were placed on a new 12-well plate in fresh Pneumacult^TM^-ALI maintenance medium. Thereafter, the exposed PBEC were transported in a portable incubator at 37 °C for analysis of bead motion induced by ciliary beating, or placed in the incubator at 37 °C and 5% CO_2_ in humidified air for recovery for 6 h or 24 h until harvesting. To control for possible impact of transporting the PBEC to another lab, exposure to a non-sterile environment, and airflow in the exposure system, we also included incubator-controlled PBEC cultures in our experiment. The experiments were conducted in triplicate per donor per exposure or control condition.

### 2.7. RNA Isolation and Real-Time Quantitative PCR

Following recovery for 6 h or 24 h, total RNA was extracted from the PBEC by lysis in TRIzol^TM^ reagent (Invitrogen^TM^, Carlsbad, CA, USA). The lysates were processed following the manufacturer’s instructions (catalog numbers 15596026 and 15596018, Invitrogen^TM^) using glycogen blue co-precipitant (Thermo Fisher Scientific). To verify the quantity and quality of the total RNA, the RNA was analyzed using the NanoDrop ND 1000 UV-vis spectrophotometer (Isogen Life Sciences, Utrecht, Netherlands). Next, total RNA (100 or 400 ng) was reverse transcribed into cDNA using an iScript^TM^ cDNA synthesis kit (Bio-Rad, Lunteren, Netherlands) including a no-reverse transcription control and a no-template control. cDNA was diluted in milliQ (1:50) and stored at −20 °C until use. Real-time quantitative PCR amplification was performed by mixing 4.4 µL of 1:50 diluted cDNA, 5 µL 2 × SensiMix^TM^ SYBR^®^ & Fluorescein Kit (Bioline, Little Clacton, UK), and 0.6 µL primers specific for genes of interest, in white LightCycler480 384 multiwell plates (Roche, Basel, Switzerland), and subsequently running a thermal cycling protocol of 10 min at 95 °C, 55 cycles of 10 s at 95 °C, 20 s at 60 °C, on the LightCycler480 machine (Roche). Supplementary Table S2 shows the list of target-specific primers used for qPCR analysis.

Qualitative analysis of the melt curves was conducted using LightCycler480 software (Roche), and quantitative gene expression analysis was performed in LinRegPCR software 2014.x (LinRegPCR v11.0. http://LinRegPCR.nl.0, accessed on 1 July 2022). The geometric mean of a combination of at least two and up to four reference genes (*ACTB*, *B2M*, *PPIA*, *RPL13A*) was calculated in GeNorm software 3.4 (Primerdesign, Austin, TX, USA). This geometric mean was used for normalization of the relevant gene-expression levels. Samples with no amplification, no plateau phase, too low Cq value, or outside 5% of the group mean were automatically excluded from the LinRegPCR analysis. In addition, qualitative analysis of melt curves and peaks revealed by the LightCycler480 software resulted in the exclusion of outliers.

### 2.8. DNA Isolation and Analysis of Mitochondrial DNA (mtDNA) Copy Number

After 24 h of recovery, PBEC were lysed in TRIzol^TM^ reagent (Invitrogen^TM^) as indicated in the previous section. Isolation of DNA was conducted following the manufacturer’s instructions (MAN0016385; catalog numbers 15596026 and 15596018, Invitrogen^TM^). The DNA pellet was dissolved in 50 µL TE buffer (10 mM tris HCI pH 8.0, 1 mM EDTA pH 8.0). To increase solubility, the samples were heated to 55 °C for 1 h and stored at 4 °C overnight. The next day, samples were centrifuged to remove insoluble materials, and supernatants were stored at −20 °C until use. The quantity and quality of the DNA was verified using the NanoDrop ND 1000 UV-vis spectrophotometer (Isogen Life Sciences, Utrecht, Netherlands). 200 ng of DNA (diluted in TE buffer) was analyzed by real-time quantitative PCR amplification using 2 × SensiMix^TM^ SYBR^®^ and Fluorescein Kit (Bioline, Netherlands; also see Section 2.7: RNA isolation and real-time quantitative PCR). Assessment of mtDNA copy numbers was performed by analyzing the ratio of mtDNA, mitochondrial encoded *MT-CO2* versus genomic DNA, and *ACTB* (Supplementary Table S2).

### 2.9. Fixation for Paraffin-Embedded Section and Immunohistochemistry Staining

PBEC inserts were harvested for immunohistochemistry staining at day 0 (undifferentiated; at moment of airlift) and day 28 (differentiated) in order to assess differentiation status. At the day of harvest, the PBEC inserts were gently washed with ice-cold HBSS (apical and basolateral). Next, 4% paraformaldehyde in PBS was added to the basolateral (1 mL) and apical (0.5 mL) sides of the insert. After at least 1.5 h incubation at room temperature, the paraformaldehyde was replaced by 70% ethanol. Fixed inserts were embedded in paraffin, cut, and stained with hematoxyline-eosine (H&E), periodic acid-Schiff (PAS; polysaccharides and mucosubstances) (Periodic acid solution 0.5% (Sigma 1004821000) and Schiff Reagent (Sigma Aldrich, 3952016-500ML)), or target-specific antibodies, as listed in Supplementary Table S3. The target-specific antibodies were employed to verify the differentiation status of the PBEC donors, basal cell and differentiation markers were stained using the antibodies P63 (nuclei), Clara cell secretory protein-16 (CC16; Clara cell protein 16), and acetylated tubulin (AcTub; present in cilia).

### 2.10. Cytotoxicity Assay

Following 6 h or 24 h of recovery after exposure, we collected the apical wash (HBSS) and basolateral medium (Pneumacult^TM^-ALI maintenance medium) in order to measure the lactate dehydrogenase (LDH) activity using the cytotoxicity detection kit (LDH Roche, USA). The cytotoxicity was analyzed within 7 days after harvesting, according to the manufacturer’s protocol with the minor adaptation of dilution of the reagents 1:1 in HBSS. The positive control reflecting maximum cytotoxicity, referred to as LDHmaximum (LDHmax), was determined by optimal lysis of an incubator-control insert of fully differentiated PBEC at the 24 h harvesting timepointh via shaking for 10 min in 2% Triton-X-100. Triplicate samples of 1/10 diluted LDHmax in Pneumacult^TM^-ALI maintenance medium were analyzed for each experiment to determine the relative LDH response. Colorimetric spectrophotometry (Molecular Devices, San Jose, CA, USA, Spectramax M2) was applied to detect cytotoxicity, after correction for the background of the medium (apical: HBSS; basolateral: Pneumacult^TM^-ALI maintenance medium), and cytotoxicity was calculated relative to the LDHmax (%).

### 2.11. Inflammatory Protein Secretion

Following the manufacturer’s protocol, with minor modifications, the LEGENDplex^TM^ Human Essential Immune Response Panel (13-plex) (BioLegend, San Diego, CA, USA) was employed to analyze the levels of 13 inflammatory proteins involved in the immune response, respectively interleukin (IL) 4, IL-2, C-X-C motif chemokine ligand (CXCL) 10 (IP-10), IL-1β, tumor necrosis factor alpha (TNF-α), C-C motif chemokine ligand (CCL2; MCP-1), IL-17A, IL-6, IL-10, interferon gamma (IFN-γ), IL-12p70, CXCL8 (IL-8), and free active transforming growth factor beta 1 (TGF-β1). Using FACS (BD FACSCanto II), these interleukins, cytokines, and chemokines were measured in the undiluted apical and basolateral supernatant of the PBEC exposed to CS or the mixture of aldehydes (6 or 24 h post-exposure). Thereafter, levels of cytokines and chemokines were calculated based on the standard curve (0.169–10.000 pg/mL).

### 2.12. Protein Isolation and Western Blotting

Following recovery for 6 h or 24 h, total protein of PBEC was isolated in 200 µL of whole cell lysis buffer (20 mM tris pH 7.4, 150 mM NaCl, 1% Nonidet P40 in MilliQ) or Pierce RIPA buffer (Thermo Fisher Scientific) including PhosSTOP phosphatase and cOmplete Mini EDTA-free protease inhibitor cocktail tablets (both Roche). Next, whole cell lysates were mixed by rotation for 30 min at 4 °C and centrifuged at 20,000× *g* for 30 min at 4 °C. Based on the determination of total protein content using the Pierce^TM^ BCA protein assay kit (catalog number 23225 and 23227, Thermo Fisher Scientific), whole cell lysates were diluted (1 µg/µL) in a final concentration of 1 × Laemmli buffer (0.25 M tris pH 6.8, 8% (*w*/*v*) sodium dodecyl sulphate, 40% (*v*/*v*) glycerol, 0.4 M dithiothreitol, 0.02% (*w*/*v*) bromophenol blue). Thereafter, lysates were boiled for 5 min at 100 °C and stored at −80 °C until analysis. The samples (10 µg of protein per lane) and corresponding protein ladders (Precision Plus Protein™ All Blue Standards #161-0373, Bio-Rad) were separated by electrophoresis (100–130 V for 1 h) on Criterion XT Precast 4–12% or 12% bis-tris gel (Bio-Rad) in 1 × MES running buffer (Bio-Rad). Next, transfer of the proteins from the gel to a 0.45 µM nitrocellulose transfer membrane (Bio-Rad) was conducted by electroblotting (Bio-Rad Criterion Blotter) (100 V for 1 h). Staining of total protein content on the blotted membrane was performed by incubation of the nitrocellulose membranes with 0.2% Ponceau S in 1% acetic acid (Sigma-Aldrich), followed by visualization using an Amersham™ Imager 600 (GE Healthcare, Eindhoven, Netherlands). Staining for the target-specific proteins started with washing off the Ponceau S staining and blocking the membranes for 1 h in 5% (*w*/*v*) non-fat dry milk (Campina, Amersfoort, Netherlands) dissolved in Tween20 tris-buffered saline (TBST; 20 mM Tris, 137 mM NaCl, 0.1% (*v*/*v*) Tween20, pH 7.6). After washing with TBST, the membranes were incubated with a target-specific primary antibody diluted in 3% (*w*/*v*) BSA (Supplementary Table S4) at 4 °C overnight. Next, a TBST wash of the membranes was followed by incubation with a horseradish-peroxidase-conjugated secondary antibody (Supplementary Table S3) diluted in 5% (*w*/*v*) non-fat dry milk in TBST for 1 h at room temperature. The membranes were subsequently washed, stained with either 1 × Supersignal West FEMTO or 0.5 × Supersignal West PICO chemiluminescent substrate (Thermo Fisher Scientific), and imaged using the Amersham™ Imager 600. Quantification of total protein content and target-specific proteins was performed on original unaltered images using Image Quant software (GE Healthcare). Total protein content was quantified using the Ponceau-S-stained images, where possible, over the entire size range of the proteins (250 kDa–10 kDa). For analysis of target-specific proteins, correction was applied for total protein loading. As some proteins of different molecular mass were analyzed on the same gel, quantification of these proteins was based on normalization with the same Ponceau S staining. The analyses using Gel I were for hexokinase 2 (HK2), dynamin 1-like (DNM1L), OXPHOS complexes including NADH ubiquinone oxidoreductase subunit B8 (NDUFB8; CI), succinate dehydrogenase complex iron sulfur subunit B (SDHB; CII), ubiquinol-cytochrome C reductase core protein 2 (UQCRC2; CIII), ATP synthase F1 subunit alpha (ATP5F1A; CV), sequestosome 1 (SQSTM1), and translocase of outer mitochondrial membrane 20 (TOMM20). Those carried out on Gel II were for parkin RBR E3 ubiquitin protein ligase (PRKN), BCL2 interacting protein 3-like (BNIP3L), BCL2 interacting protein 3 (BNIP3), FUN14 domain containing 1 (FUNDC1), PTEN induced kinase 1 (PINK1), and microtubule-associated protein 1 light chain 3 beta (MAP1LC3B). Analysis on Gel III included detection of peroxisome proliferator-activated receptor gamma, coactivator 1 alpha (PPARGC1A), nuclear respiratory factor 1 (NRF1), GABA type A receptor associated protein like 1 (GABARAPL1), and estrogen-related receptor alpha (ESRRA).

Representative Western blot images in the figures of this manuscript have been adjusted for brightness and contrast equally throughout the picture. Moreover, the 85 kDa Ponceau S bands included in the figures are representative of the whole Ponceau S staining, and the selected target-specific band shown for one replicate from one donor per experiment is reflective of the mean changes in all donors as quantified in the corresponding graph.

### 2.13. Analysis of Bead Motion Induced by Ciliary Beating

Immediately after exposure, PBEC were transported in a portable incubator at 37 °C for analysis of bead motion induced by ciliary beating. The exposed PBEC were placed under the microscope (Leica Dmi8), and maintained at 37 °C and 5% CO_2_ in humidified air. Directly before video was recorded, 10 µL of 2.1 µM beads (RED PSRF 2.1 µM 2.5% PSFR3961A-1219; Magsphere) diluted 1:50,000 in Pneumacult^TM^-ALI maintenance medium were added to the apical side of the exposed PBEC in the middle of the well. Recording of the bead motion started circa 5–10 min after addition of the beads, to facilitate spreading of the beads over the well and their movement in a flow induced by ciliary beating. Videos were acquired from 30 min until 2.5 h post-exposure using a LEICA DFC7000 GT camera with a 4× objective, along with LAS X 3.4.2 software. Videos were recorded with a frame interval of 0.33 s for 20–60 s.

Automatic tracking of the beads was conducted in ImageJ version 1.53 [56] using the MTrackJ plugin for motion tracking and analysis [57]. First, we selected videos recording the motion of the beads at the edge of the well, to retain a consistent position because flow speed varied according to position within the well. Moreover, beads were followed outside the mucus, because flow speed differed between inside and outside the mucus. For each exposure condition (CS, mixture of aldehydes, or air), one to three videos per well were selected for each donor, where available. Videos were loaded into ImageJ, and scale was modified to 0.2065 pixels/µM. Beads located as close as possible to the edge of the well and following a smooth path were selected for tracking (Figure S9A). Within one video (i.e., one well), 5–6 beads were tracked for 2.97–5.00 s (i.e., 9–16 frames) (see example in Figure S9B). Subsequently, mean distance per second was calculated for each exposure condition for each donor, according to tracked beads from 1–3 videos, respectively resulting in 5–12 tracked beads per donor exposed to air or CS and 6–23 tracked beads per donor exposed to air or aldehydes. In summary, results for total numbers of tracked beads across all donors were air: 29 vs. CS: 24, and air: 18 vs. aldehydes: 41.

### 2.14. Statistical Analysis

Statistical analysis and data graphing were carried out using GraphPad Prism 8.0 software (USA). The experiments were conducted in triplicate for each independent donor under each condition. The mean values for each donor are represented in the bar charts by open circles (donor 1), triangles (donor 2), squares (donor 3), or diamonds (donor 4). The data are presented as mean fold change (FC) compared to air control ± standard error of the mean (s.e.m.). Statistical differences between air vs. CS and air vs. mixture of aldehydes were tested using a two-tailed paired parametric *t*-test. Statistical significances were indicated as *p*-values below 0.05 (* *p* < 0.05) or below 0.01 (** *p* < 0.01).

## 3. Results

### 3.1. Validation and Characterization of Differentiation of PBEC at ALI

To validate and characterize our model of differentiation of PBEC at the ALI, we analyzed mRNA expression of markers associated with basal and luminal cells during the differentiation of donors. Basal epithelial cells were identified by markers *KRT5*, *KRT14*, and the nuclear *TP63*. Moreover, club cells were identified by *SCGB1A1*, ciliated cells with *FOXJ1*, and goblet cells with *MUC5AC*. 

As depicted in Supplementary Figure S3A,B, mRNA levels of basal epithelial cell markers decreased, while increased transcript abundance of markers associated with cilia, club, and goblet cells were observed in fully differentiated cultures (day 28) compared with non-differentiated cultures (day 0 at moment of airlift). To further verify differentiation status of the PBEC donors (14, 21, 28 days post-airlift), we conducted H&E staining as well as immunohistochemistry staining of nuclei, Clara cell protein 16, polysaccharides, mucosubstances, and cilia. The presence of a pseudostratified epithelium as well as identification of markers associated with specific cell types were confirmed from one representative donor at 28 days post-airlift, visualized in Figure S3C,D. Based on these findings, PBEC cultures at the ALI differentiated for at least 28 days included several cell types representing the pseudostratified epithelium.

In addition, to monitor monolayer integrity during differentiation, TEER was measured in all PBEC cultures from all donors at different timepoints. As depicted in Supplementary Figure S4, the TEER (Ω·cm^2^) of all PBEC donors increased over time and stabilized at 28 days post-airlift, indicating a confluent intact monolayer before exposure to CS and aldehydes.

### 3.2. No Impact of CS or Aldehydes Exposure on Cell Viability and Monolayer Integrity

After validation and characterization of differentiation of PBEC at the ALI, we assessed the effects of single exposure to CS or aldehydes on cell viability and monolayer integrity. Exposure to CS or aldehydes did not impact cell viability 6 h or 24 h post-exposure (Supplementary Figure S5A,B). Moreover, immediately (±3 h) after exposure, permeability of PBEC cultures assessed by the FITC-dextran assay was negligible, indicating the integrity of the monolayer (Supplementary Figure S5C). In addition, we took into account the potential stressful impact of the exposure system (e.g., transport, air-flow) by including incubator controls in our study. In general, no pronounced impact of dynamic exposure to air was observed for any of the analyzed markers (incubator versus air control), except for small but significant increases in secretion levels of IL-8, IL-6, and CCL-2 in apical supernatants of PBEC after 6 h of exposure (data not shown). Moreover, small decreases in transcript levels of genes encoding for IL-8, autophagy, and fission or fusion were observed in response to air-flow (data not shown). These findings support a negligible impact of air-flow per se on our cultures.

### 3.3. CS Exposure Affects Inflammatory Protein Production and Antioxidant Gene Expression

**Figure 1:**
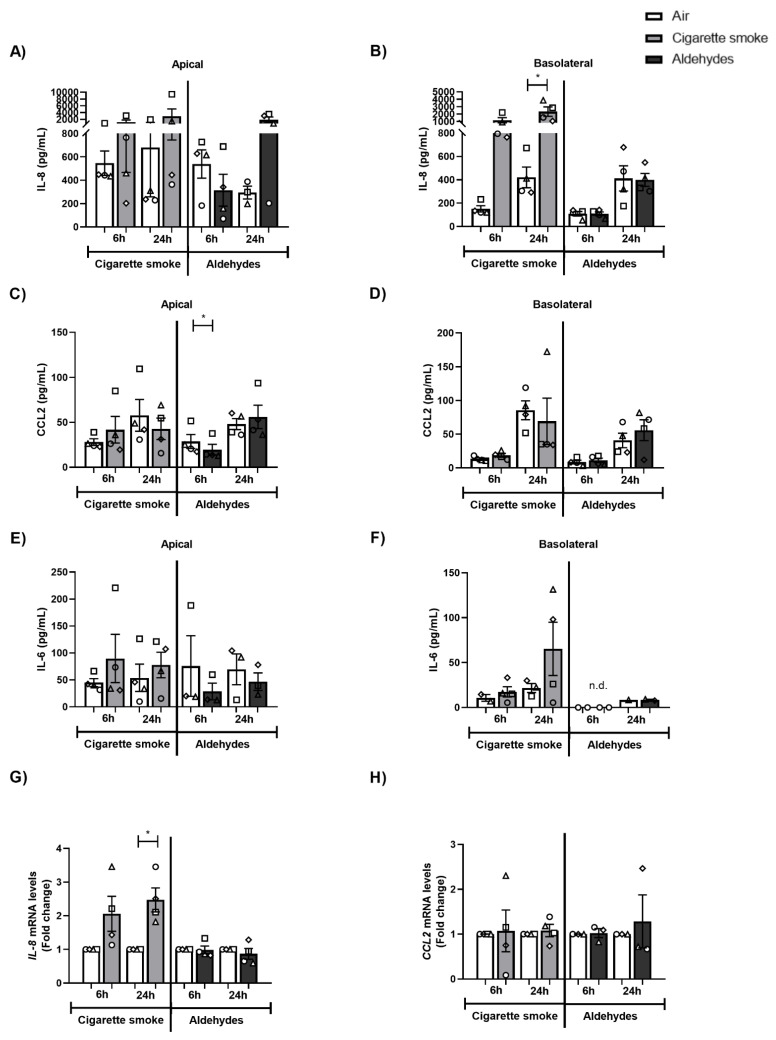
Impact of CS exposure on inflammatory proteins and inflammatory gene expression. Differentiated human primary bronchial epithelial cells (PBEC) from non-COPD subjects (*n* = 2–4 donors) were exposed to smoke of one Marlboro Red cigarette (CS), or a mixture of acetaldehyde, acrolein, and formaldehyde (at concentrations equivalent to one cigarette), or air (control), under a continuous flow system using a puff-like exposure protocol. Following recovery for 6 h or 24 h, supernatants and whole cell lysates were harvested. Inflammatory protein levels: (**A**,**B**) IL-8, (**C**,**D**) CCL-2, and (**E**,**F**) IL-6 in apical and basolateral supernatants were analyzed. Transcript levels of inflammatory markers (**G**) *IL-8* and (**H**) *CCL-2* in whole cell lysates were analyzed. Data are presented as mean fold change compared to air control ± s.e.m. The mean values of biological triplicate results per independent donor are represented by open circles, triangles, squares, or diamonds. Statistical differences between CS vs. air and the mixture of aldehydes vs. air were tested using a two-tailed paired parametric *t*-test. Statistical significance is indicated as * *p* < 0.05 vs. air (control).

Next, we investigated the production of several inflammatory proteins (interleukins, cytokines, chemokines) as well as mRNA expression of inflammatory genes and antioxidant enzymes in response to CS or aldehydes. Although IL-8, IL-6, and CCL2 were detectable in both apical and basolateral media at baseline, only IL-8 levels were found to be significantly increased in the basolateral medium of CS-exposed PBEC (Figure 1A–F). Moreover, we observed elevated gene expression of Il-8 in response to CS exposure (Figure 1G). In general, no profound changes in response to aldehydes were observed in these inflammatory proteins, nor in terms of inflammatory gene expression, except for decreased CCL2 levels in apical medium (Figure 1C). Secretion of IL-4, IL-2, CXCL10, IL-1β, TNF-α, IL-17A, IL-10, IFN-γ, IL-12p7, and free active TGF-β1 was in general non-detectable (data not shown).

**Figure 2:**
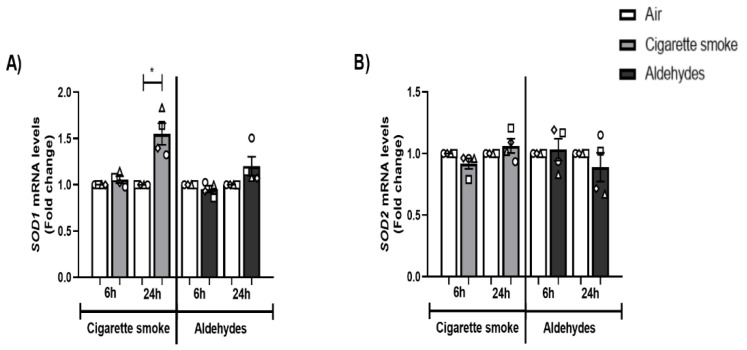
CS exposure affects expression of antioxidant genes. Differentiated human primary bronchial epithelial cells (PBEC) from non-COPD subjects (*n* = 4 donors) were exposed to smoke of one Marlboro Red cigarette (CS) or a mixture of acetaldehyde, acrolein, and formaldehyde (at concentrations equivalent to one cigarette) or air (control), under a continuous flow system using a puff-like exposure protocol. Following recovery for 6 h or 24 h, whole cell lysates were harvested for transcript analysis of oxidative stress markers (**A**) *SOD1* and (**B**) *SOD2*. Data are presented as mean fold change compared to air control ± s.e.m. The mean values of biological triplicate results per independent donor are represented by open circles, triangles, squares, or diamonds. Statistical differences between CS vs. air and a mixture of aldehydes vs. air were tested using a two-tailed paired parametric *t*-test. Statistical significance is indicated as * *p* < 0.05 vs. air (control).

With respect to the expression of antioxidant genes, the transcript levels of *SOD1* were significantly elevated 24 h post-CS exposure, while the expression of *SOD2* was unaltered. Exposure to the aldehydes mixture did not alter antioxidant gene expression (Figure 2A,B).

In conclusion, although CS exposure affected the secretion levels of IL-8 as well as the transcript abundance of inflammatory and antioxidant genes, aldehydes had a minor impact. 

### 3.4. Altered Abundance of Molecules Associated with Autophagy Following CS or Aldehyde Exposure

**Figure 3:**
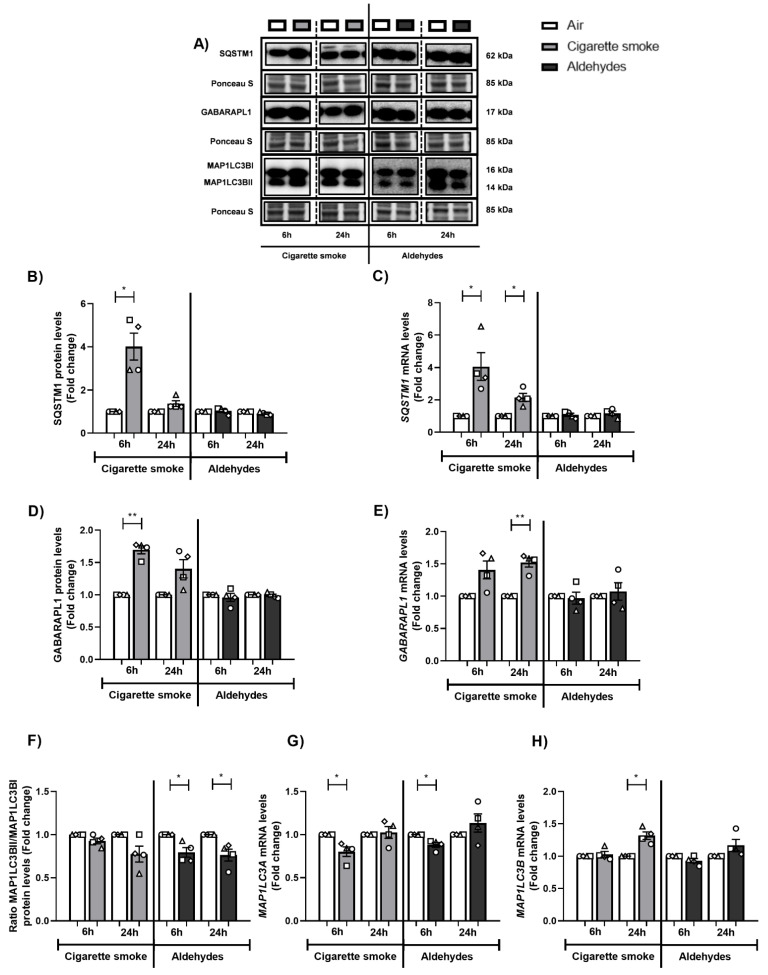
Changes in the abundance of autophagic components in response to exposure to CS or aldehydes. Differentiated human primary bronchial epithelial cells (PBEC) from non-COPD subjects (*n* = 4 donors) were exposed to smoke of one Marlboro Red cigarette (CS), or a mixture of acetaldehyde, acrolein, and formaldehyde (at concentrations equivalent to one cigarette), or air (control), in a continuous flow system using a puff-like exposure protocol. Following recovery for 6 h or 24 h, whole cell lysates were harvested for analysis of the (**A**,**B**,**D**,**F**) protein and (**C**,**E**,**G**,**H**) transcript abundance of constituents associated with autophagy, respectively SQSTM1, GABARAPL1, ratio MAP1LC3BII/I, and MAP1LC3A and MAP1LC3B. Representative Western blot images are shown of one replicate of one donor per experiment, reflective of the changes in all donors as quantified in the corresponding graph. Data are presented as mean fold change compared to air control ± s.e.m. The mean values from biological triplicates per independent donor are represented by open circles, triangles, squares, or diamonds. Statistical differences between CS vs. air and the mixture of aldehydes vs. air were tested using a two-tailed paired parametric *t*-test. Statistical significance is indicated as * *p* < 0.05 and ** *p* < 0.01 vs. air (control).

The autophagosomal pathway has an important role clearing (sub)cellular damage, and previous in vitro studies suggested that acute exposure of PBEC to CS resulted in increased abundance of autophagic components [50]. We therefore assessed the expression of specific autophagy proteins in response to CS or aldehydes. After 6 h or 24 h of recovery following exposure to CS, protein and transcript levels of SQSTM1 and GABARAPL1 were significantly increased (Figure 3A–E). These results were not replicated in response to aldehyde exposure. The protein ratio of MAP1LC3BII/MAP1LC3BI did not change after exposure to CS, while this protein ratio was decreased in PBEC exposed to aldehydes (Figure 3A,F). Exposure to CS as well as to aldehydes resulted in decreased *MAP1LC3A* and/or increased *MAP1LC3B* mRNA abundance (Figure 3G,H).

### 3.5. Exposure to CS Decreases Expression of Components of the Mitophagy Machinery

Because exposure to aldehydes or CS is associated with alterations in the abundance of specific autophagy proteins, and these proteins are critical for facilitating the degradation of damaged or dysfunctional mitochondria (i.e., mitophagy), we further assessed the effect of smoking-associated aldehyde exposure on the abundance of constituents within the mitophagy machinery.

**Figure 4:**
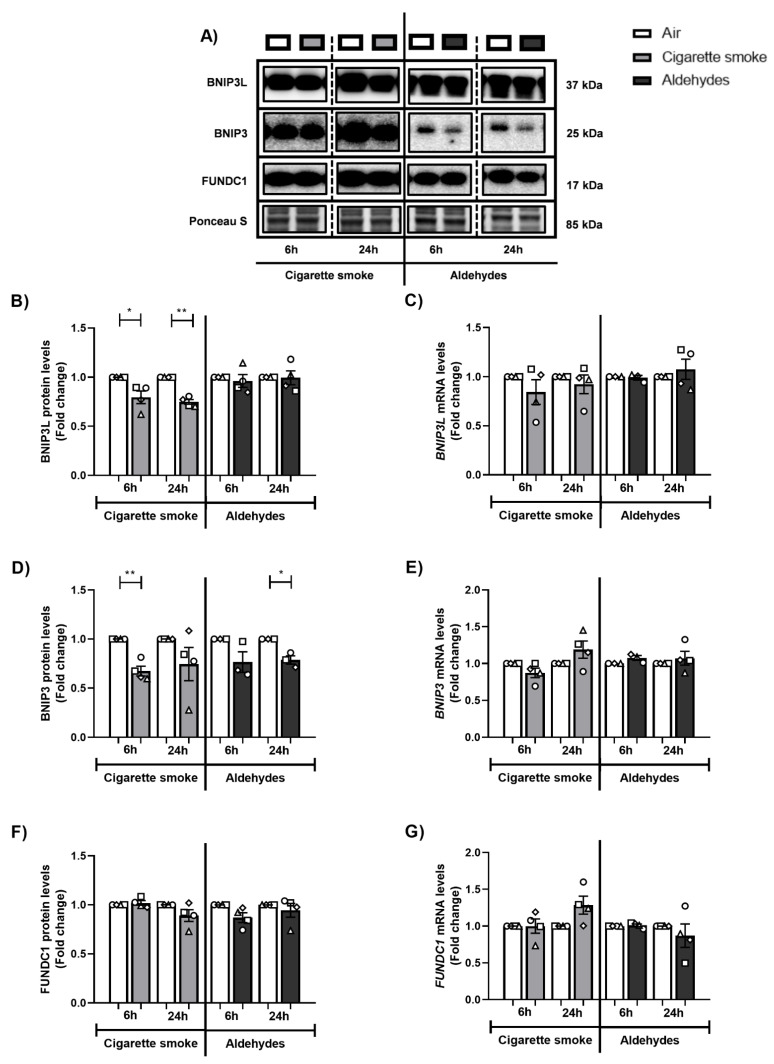
Decreased protein levels of markers associated with receptor-mediated mitophagy after CS exposure. Differentiated human primary bronchial epithelial cells (PBEC) from non-COPD subjects (*n* = 3–4 donors) were exposed to smoke of one Marlboro Red cigarette (CS), or a mixture of acetaldehyde, acrolein, and formaldehyde (at concentrations equivalent to one cigarette), or air (control), under a continuous flow system using a puff-like exposure protocol. After recovery for 6 h or 24 h, whole cell lysates were harvested for analysis of (**A**,**B**,**D**,**F**) protein and (**C**,**E**,**G**) transcript abundance of key regulators involved in receptor-mediated mitophagy: BNIP3L, BNIP3, and FUNDC1. Representative Western blot images are shown for one replicate of one donor per experiment, reflective of the changes in all donors as quantified in the corresponding graph. Data are presented as mean fold change compared to air control ± s.e.m. The mean values of biological triplicates for each independent donor are represented by open circles, triangles, squares, or diamonds. Statistical differences between CS vs. air and the mixture of aldehydes vs. air were tested using a two-tailed paired parametric *t*-test. Statistical significance is indicated as * *p* < 0.05 and ** *p* < 0.01 vs. air (control).

Firstly, we investigated the impact of CS or the mixture of aldehydes on the abundance of proteins involved in receptor-mediated mitophagy. As shown in Figure 4, significantly decreased protein levels of BNIP3L and BNIP3 were observed following CS exposure, while the impact of aldehydes was less pronounced. Moreover, protein levels of FUNDC1 as well as mRNA levels of markers of receptor-mitophagy did not significantly change following CS or aldehyde exposure.

Secondly, the abundance of key regulators involved in ubiquitin-mediated mitophagy was examined. As depicted in Supplementary Figure S6, exposure to CS or aldehydes resulted in decreased transcript levels of *PINK1* after 6 h of recovery, while other markers related to ubiquitin-mediated mitophagy were unaltered both at protein and mRNA level. 

In summary, although CS had a significant impact on the abundance of key regulators involved in autophagy and mitophagy, the effect of aldehydes on constituents associated with these processes was less pronounced.

### 3.6. Alterations in the Expression of Proteins and Genes Involved in Mitochondrial Biogenesis in Response to CS

**Figure 5:**
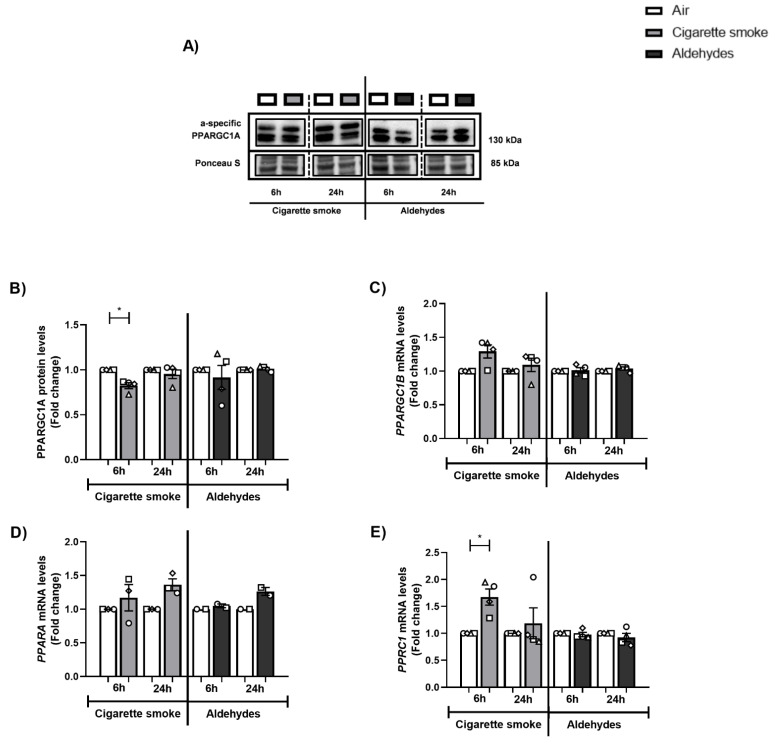
Altered abundance of constituents controlling mitochondrial biogenesis following CS exposure. Differentiated human primary bronchial epithelial cells (PBEC) from non-COPD subjects (*n* = 3–4 donors) were exposed to smoke of one Marlboro Red cigarette (CS), or a mixture of acetaldehyde, acrolein, and formaldehyde (at concentrations equivalent to one cigarette), or air (control), under a continuous flow system using a puff-like exposure protocol. Following recovery for 6 h or 24 h, whole cell lysates were harvested and (**A**,**B**) protein levels of PPARGC1A, as well as (**C**–**E**) transcript levels of PPARGC1 molecules: *PPARGC1B*, *PPARA*, *PPRC1* were analyzed. Representative Western blot images are shown for one replicate of one donor per experiment, reflective of the changes in all donors as quantified in the corresponding graph. Data are presented as mean fold change compared to air control ± s.e.m. The mean values of biological triplicates for each independent donor are represented by open circles, triangles, squares, or diamonds. Statistical differences between CS vs. air and the mixture of aldehydes vs. air were tested using a two-tailed paired parametric *t*-test. Statistical significance is indicated as * *p* < 0.05 vs. air (control).

**Figure 6:**
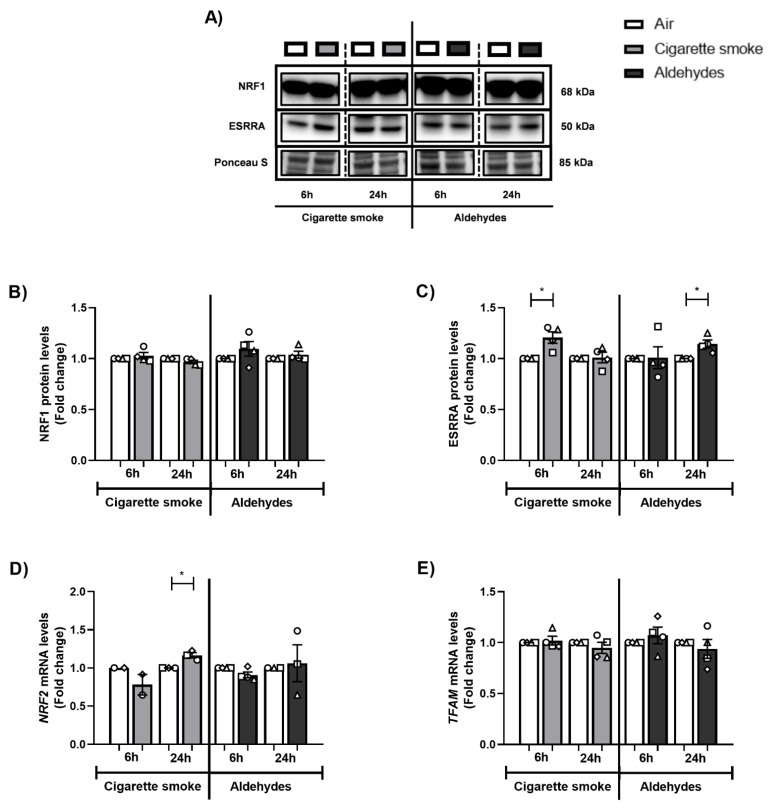
CS exposure results in changes in the abundance of PPARGC1-coactivated transcription factors. Differentiated human primary bronchial epithelial cells (PBEC) from non-COPD subjects (*n* = 2–4 donors) were exposed to smoke of one Marlboro Red cigarette (CS), or a mixture of acetaldehyde, acrolein, and formaldehyde (at concentrations equivalent to one cigarette), or air (control), in a continuous flow system using a puff-like exposure protocol. After recovery for 6 h or 24 h, whole cell lysates were harvested to analyze (**A**) protein levels of (**B**) NRF1 and (**C**) ESSRA. Representative Western blot images are shown for one replicate from one donor per experiment, reflective of the changes in all donors as quantified in the corresponding graph. Transcript levels of (**D**) *NRF2* and (**E**) *TFAM* were analyzed. Data are presented as mean fold change compared to air control ± s.e.m. The mean values of biological triplicates per independent donor are represented by open circles, triangles, squares, or diamonds. Statistical differences between CS vs. air and a mixture of aldehydes vs. air were tested using a two-tailed paired parametric *t*-test. Statistical significance is indicated as * *p* < 0.05 vs. air (control).

Next, we investigated the abundance of key transcription factors involved in mitochondrial biogenesis, specifically molecules co-activated by PPARGC1. Although we observed decreased protein abundance of PPARGC1A, protein levels of ESRRA and the mRNA expression of other coactivators in this pathway (*PPRC1, NRF2*) were upregulated in response to CS (Figure 5 and Figure 6). The abundance of PPARGC1 transcription factors was in general unchanged upon aldehyde exposure, with the exception of significantly elevated ESRRA protein levels 24 h post-exposure to aldehydes.

These data indicate that CS exposure especially resulted in altered expression of constituents controlling mitochondrial biogenesis pathways in PBEC, while the aldehydes present in CS had a minimal impact on the abundance of mitochondrial biogenesis transcription factors.

### 3.7. CS and Aldehydes Affect the Abundance of Mitochondrial Fission and Fusion Regulators

In addition to mitophagy and mitochondrial biogenesis, mitochondrial fission and fusion are essential processes in mitochondrial quality control and homeostasis. To study whether the CS- and/or aldehyde-induced changes in the abundance of regulators involved in mitophagy and mitochondrial biogenesis were accompanied by disruption of mitochondrial dynamics, we examined the abundance of fission- and fusion-associated proteins and genes following CS and aldehyde exposure.

**Figure 7:**
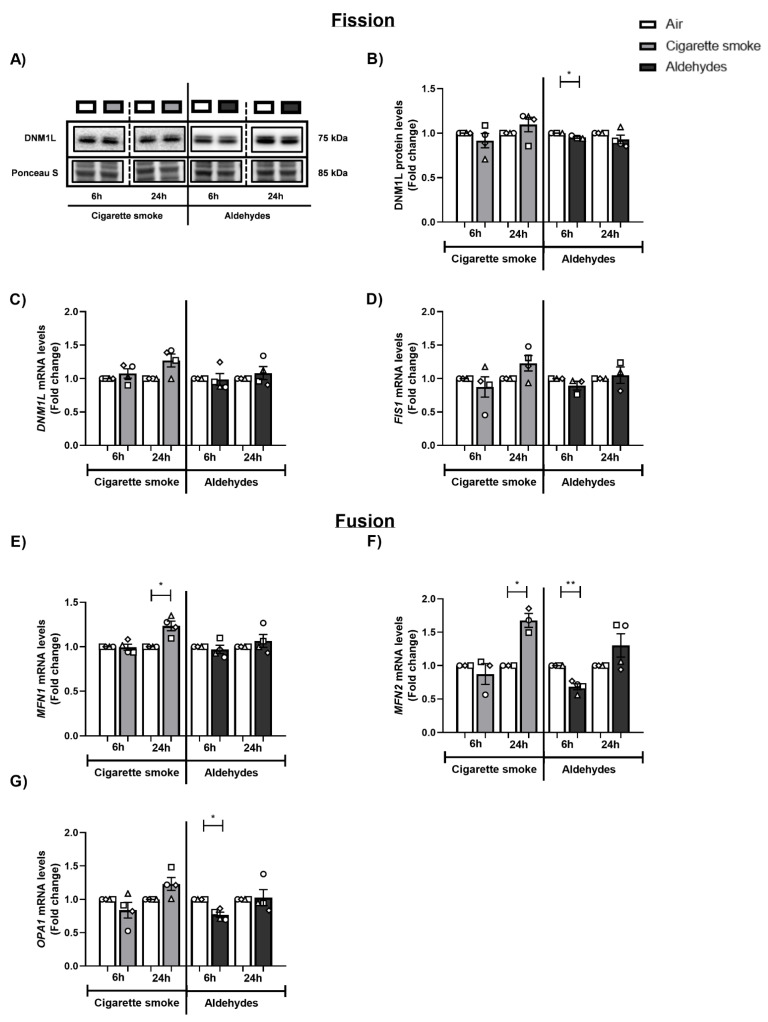
Abundance of mitochondrial fission- and fusion-associated markers is altered after exposure to CS or aldehydes. Differentiated human primary bronchial epithelial cells (PBEC) from non-COPD subjects (*n* = 3–4 donors) were exposed to smoke of one Marlboro Red cigarette (CS), or a mixture of acetaldehyde, acrolein, and formaldehyde (at concentrations equivalent to one cigarette), or air (control), under a continuous flow system using a puff-like exposure protocol. After recovery for 6 h or 24 h, whole cell lysates were harvested for analysis of fission-associated markers, (**A**,**B**) DNM1L protein as well as (**C**) *DNM1L* and (**D**) *FIS1* transcript levels. Representative Western blot images are shown for one replicate from one donor per experiment, reflective of the changes in all donors as quantified in the corresponding graph. In addition, the expression of fusion-associated genes (**E**) *MFN1*, (**F**) *MFN2*, and (**G**) *OPA1* was analyzed. Data are presented as mean fold change compared to air control ± s.e.m. The mean values of biological triplicates for each independent donor are represented by open circles, triangles, squares, or diamonds. Statistical differences between CS vs. air and the mixture of aldehydes vs. air were tested using a two-tailed paired parametric *t*-test. Statistical significance is indicated as * *p* < 0.05 and ** *p* < 0.01 vs. air (control).

Regarding the response of fission-associated markers, we observed minimal decreases in DNM1L protein levels following aldehyde exposure (6 h) (Figure 7A,B). Transcript level analysis of the fission genes *DNM1L* and *FIS1* showed no differences following exposure to CS or aldehydes, compared to air control (Figure 7C,D). A similar pattern was observed when investigating the expression of fusion-associated genes. mRNA levels of *MFN1* and *MFN2* were increased 24 h post-exposure to CS, while *MFN2* and *OPA1* mRNA levels were significantly decreased in response to aldehydes (6 h) (Figure 7E–G).

In summary, we observed that exposure to CS resulted in increased transcript abundance of fusion-associated markers 24 h post-stimulation, while aldehyde exposure resulted in a transient decrease in expression of these dynamic mitochondrial fission and fusion markers.

### 3.8. Minor Changes in the Abundance of Subunits of Oxidative Phosphorylation Complexes in Response to Smoking-Associated Aldehyde Exposure

Next, we assessed whether exposure to CS or aldehydes affected the abundance of constituents of the electron transport chain. Protein levels of a complex I subunit were decreased following exposure to CS (24 h), which was not replicated upon aldehyde exposure (Figure S7A,B). Additionally, protein abundance levels of investigated subunits of complexes II, III, and V were unaltered after exposure (Figure S7A,C–E). Interestingly, transcript abundance of a subunit of complex I (*NDUFB3*) was not changed, while mRNA levels of complex III (*CYC1*) were elevated after aldehyde exposure (Figure S8). In addition, exposure to the mixture of aldehydes resulted in a slight decrease in protein abundance of the outer mitochondrial membrane receptor TOMM20 (Figure S7A,F). Assessment of mtDNA copy numbers showed no differences between CS or aldehyde exposure vs. air control (Figure S7G).

### 3.9. CS or Aldehyde Exposure Induced Expression of Genes Involved in Glucose Metabolism

**Figure 8:**
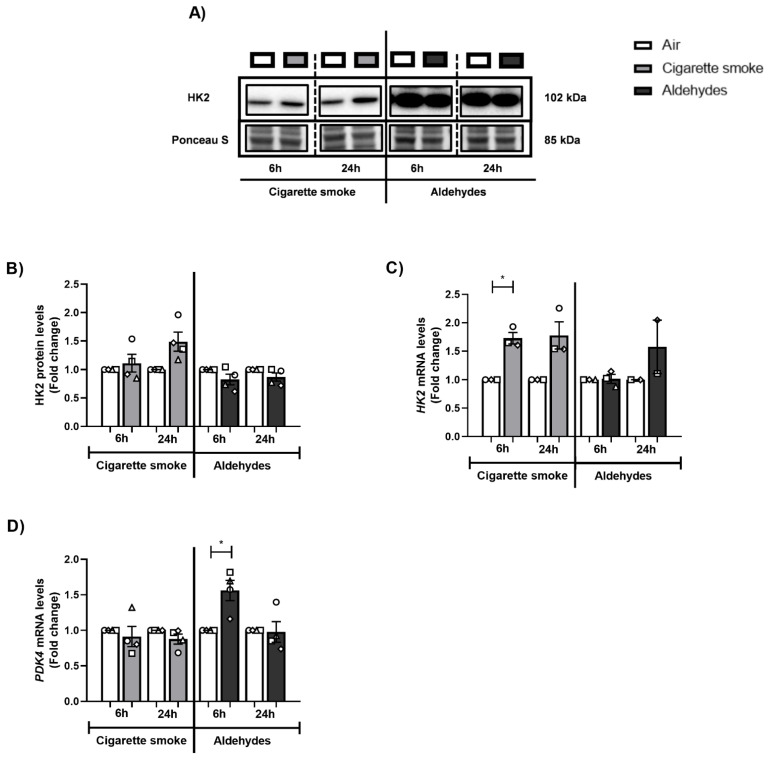
Increased abundance of genes associated with glucose metabolism upon CS or aldehyde exposure. Differentiated human primary bronchial epithelial cells (PBEC) from non-COPD subjects (*n* = 2–4 donors) were exposed to smoke of one Marlboro Red cigarette (CS), or a mixture of acetaldehyde, acrolein, and formaldehyde (at concentrations equivalent to one cigarette), or air (control), under a continuous flow system using a puff-like exposure protocol. After recovery for 6 h or 24 h, whole cell lysates were harvested to analyze (**A**,**B**) protein levels of HK2. Representative Western blot images are shown for one replicate from one donor per experiment, reflective of the changes in all donors as quantified in the corresponding graph. Transcript levels of (**C**) *HK2* and (**D**) *PDK4* were analyzed. Data are presented as mean fold change compared to air control ± s.e.m. The mean values of biological triplicates for each independent donor are represented by open circles, triangles, squares, or diamonds. Statistical differences between CS vs. air and the mixture of aldehydes vs. air were tested using a two-tailed paired parametric *t*-test. Statistical significance is indicated as * *p* < 0.05 vs. air (control).

Considering the observed impact on the molecular mechanisms controlling mitochondrial function, we also assessed the abundance of key molecules involved in other metabolic processes (i.e., glycolysis). CS exposure increased transcript abundance of the glycolytic enzyme HK2 (6 h), while protein abundance was unaltered (Figure 8A–C). This effect was absent in response to aldehydes. Moreover, *PDK4* expression, which regulates the influx of pyruvate into the mitochondria, increased after aldehyde exposure (6 h) (Figure 8D).

### 3.10. The Expression of Basal Epithelial Cell Marker KRT5 Is Decreased after CS Exposure

**Figure 9:**
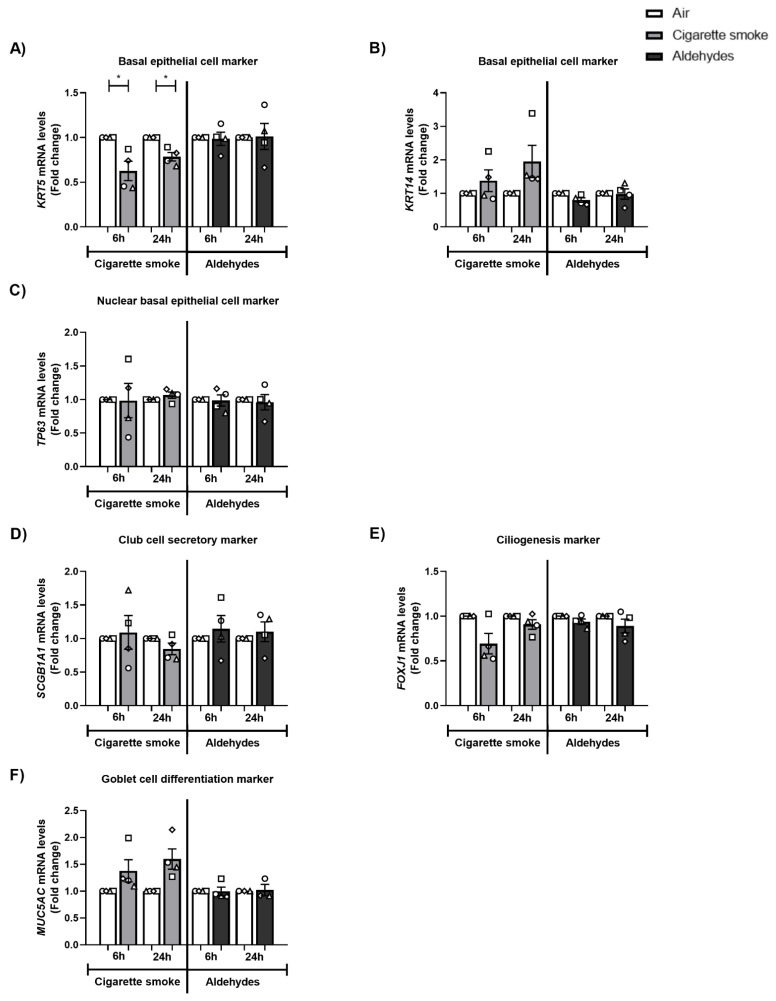
Transcript levels of basal epithelial cell marker *KRT5* are decreased in response to CS exposure. Differentiated human primary bronchial epithelial cells (PBEC) from non-COPD subjects (*n* = 4 donors) were exposed to smoke of one Marlboro Red cigarette (CS), or a mixture of acetaldehyde, acrolein, and formaldehyde (at concentrations equivalent to one cigarette), or air (control), under a continuous flow system using a puff-like exposure protocol. After recovery for 6 h or 24 h, whole cell lysates were harvested to analyze transcript levels of basal epithelial cell markers (**A**) *KRT5*, (**B**) *KRT14*, and (**C**) nuclear basal epithelial cell marker *TP63*. Expression levels were analyzed for (**D**) club cell secretory marker *SCGB1A1*, (**E**) ciliogenesis marker *FOXJ1*, and (**F**) goblet cell differentiation marker *MUC5AC*. Data are presented as mean fold change compared to air control ± s.e.m. The mean values of biological triplicates for each independent donor are represented by open circles, triangles, squares, or diamonds. Statistical differences between CS vs. air and the mixture of aldehydes vs. air were tested using a two-tailed paired parametric *t*-test. Statistical significance is indicated as * *p* < 0.05 vs. air (control).

As depicted in Figure 9A–C, CS exposure resulted in decreased expression of *KRT5* while other basal epithelial cell markers (*KRT14* and *TP63*) did not change in response to CS. These alterations in the expression of cell-specific markers were not replicated in response to the mixture of aldehydes. No pronounced impacts of CS or aldehydes were detected for club cell marker *SCGB1A1*, ciliogenesis marker *FOXJ1*, or goblet cell differentiation marker *MUC5AC* (Figure 9D–F).

### 3.11. Impact of CS or Aldehyde Exposure on Cilia Bead Flow

Because mitochondria are essential for the proper function of epithelial cells of the airways (e.g., ciliary function and mucus production), we next investigated the impact of CS-associated aldehyde exposure on the motions of bead in a flow induced by cilia beating. As shown in Supplementary Figure S9, no differences were observed in the distances travelled per second by beads in CS- or aldehyde-exposed cells compared with air. 

## 4. Discussion

In this study, CS exposure resulted in increased IL-8 secretion as well as elevated transcript levels of antioxidant and inflammatory genes. Moreover, the molecular regulation of mitochondrial quality-control processes was disrupted in response to CS exposure, mainly at the level of autophagy and mitophagy. Furthermore, transcript levels of basal epithelial cell marker *KRT5* declined in response to CS. CS-mediated responses were minimally reproduced in PBEC cultures exposed to a mixture of aldehydes.

Literature describing inflammation and oxidative stress in response to CS or aldehydes (acetaldehyde, acrolein, and formaldehyde) tested using in vivo and in vitro airway models is abundant [8,9,58,59,60,61]. We observed that only CS exposure induced an inflammatory response and antioxidant gene expression, whereas the response to aldehydes was less pronounced. It could be suggested that our use of a single exposure to aldehydes at high peak concentrations may have been insufficient to induce an inflammatory response or significantly alter cellular (anti-)oxidant status. In this context, it is important to note that we only measured antioxidant gene expression and did not directly assess (anti-)oxidant status.

Studies investigating the impact of CS exposure on the regulation of mitochondrial quality control, content, and metabolism in differentiated PBEC models, using a continuous flow system combined with a puff-like exposure regime such as we deployed, are non-existent in the literature. However, several studies using CS exposure on cell lines or undifferentiated human primary airway epithelial cells have shown that CS or CS extract (CSE) induced autophagy and mitophagy [31,33,37,38,39,40,41,43,44,45,46]. This is in line with our previous findings in whole CS-exposed differentiated PBEC [50]. Collectively, the CS-mediated autophagy/mitophagy response that we (and others) observed can be interpreted as a cellular response in an effort to clear up CS-induced damage to specific proteins, organelles, or other macromolecules. Indeed, it has previously been shown that CS causes damage to mitochondria that manifests as aberrant mitochondrial morphology, observed in experimental smoke-exposure models in vivo and in vitro [28,29,30,31,32,33,34,35,36,37], and mitochondrial dysfunction (i.e., impaired respiration) [34]. Assessment of cellular respiration in 3D cultures is challenging and was not the primary aim of this research, so we did not include these measurements in the current study. However, it is possible to measure cell respiration in 3D cultures, as described in previous publications [62,63].

An interesting observation made in our study was that the abundance of autophagy proteins drastically increased, whereas mitophagy proteins moderately decreased after CS exposure. In the case of autophagy, this might be explained in several ways. One possibility would be a blocked autophagy flux (i.e., autophagosome and associated proteins fail to be degraded, and thus accumulate). However, we also observed increased autophagy mRNA levels suggestive of another possible explanation, namely an activation of autophagy in response to damage. These two theories have been described previously, and conflicting experimental data have been reported regarding the contribution of increased or blocked autophagy as injurious or protective for the airways and the corresponding development of disease [64,65,66,67,68]. It should be noted that an obvious limitation of the current study is that we did not directly assess mitophagy or mitochondrial dynamics, which can be achieved, for example, by use of confocal microscopy and specific fluorescent probes [69]. Future research implementing these microscopy techniques is necessary to assess the contribution of (blocked) autophagy in response to CS exposure in epithelial cells of the airway.

In contrast to the results for CS, we observed that aldehydes minimally affected the abundance of autophagy or mitophagy constituents. Previous studies, although limited and carried out in non-primary human lung cells, demonstrated that aldehydes (such as acrolein and also formaldehyde) can stimulate autophagy to a similar extent as smoke [70,71,72]. Nevertheless, it is difficult to compare those findings with our results, due to variations in dose (25 µM up to 10 ppm), exposure regimes (gaseous inhalation vs. exposure in solutions), and cell types analyzed (rat lung homogenates vs. human airway cell lines).

With regard to mitochondrial biogenesis, we observed in response to CS decreased protein levels of PPARGC1A and increased transcript levels of multiple molecules involved in mitochondrial biogenesis. However, transcript levels of Tfam, responsible for transcription of the mitochondrial genome and a key regulator of mitochondrial biogenesis, were unaltered, which is in line with findings in BEAS-2B cells exposed to CSE [73]. These findings suggest a (partly) compensatory cellular response in PBECs to counteract potential damage-induced breakdown of mitochondria. Previous in vitro PBEC studies reported a contrary impact of CS exposure on transcript levels of some regulators of the PPARGC1 network [50,74]. Discrepant findings may well be explained by differences in time, dosage, and mode of exposure. In contrast to CS, aldehydes again had no profound impact on the mitochondrial biogenesis-associated molecules that we investigated in our study. Previous research reported decreased expression of constituents of the mitochondrial biogenesis machinery in response to acrolein, in cells of the lung in vitro and in lung tissue in vivo [71,75]. The fact that we did not observe these responses to aldehydes in our model may suggest that this response is limited to certain cell types in the airways, not including cells from the bronchial epithelial layer. To our knowledge, the impact of CS-associated acetaldehyde and formaldehyde on the regulation of mitochondrial biogenesis in cells of the airways has never previously been studied.

With regard to mitochondrial dynamics, we observed that exposure to CS resulted in increased transcript abundance of fusion-associated markers 24 h post-stimulation, while a transient decrease in expression of fission and fusion markers was observed in response to aldehydes. This is partly in line with our previously published data for differentiated PBEC cultured at the ALI and exposed to whole CS [50]. Moreover, other studies in vivo and in vitro have shown that acrolein exposure resulted in increased (transcriptional regulation of) mitochondrial fission [70,71], and smoke exposure resulted in mitochondrial fragmentation with increased fission and decreased fusion [30,33,34,41,44,45,76] in cells of the airways. Discrepancies have also been reported in the literature after smoke exposure in lung cells [31,38,43,77]. These conflicting findings can be explained by the various exposure models used, as well as the dynamic character of the fission and fusion flux. Interestingly, the observation of increased abundance of fusion proteins upon CS exposure is compatible with changes in the abundance of regulatory molecules controlling mitochondrial biogenesis and indications for increased autophagy, which together may be interpreted as stimulated mitochondrial turnover (i.e., increased degradation of mitochondria and increased generation of new mitochondria) after CS exposure. Again, in the case of our study, the absence of direct microscopic assessment of mitochondrial dynamics limits our conclusions in this respect.

Although CS-induced mitochondrial dysfunction has been described in airway epithelial cells in vivo [29] and in vitro [34], we observed no marked changes in the abundance of subunits of oxidative phosphorylation complexes or mtDNA copy numbers following CS exposure. These findings are in line with our previous study investigating the impact of smoke exposure in various PBEC models [50]. Interestingly, one previous study reported CSE-induced release of extracellular vesicles containing mitochondria in a bronchial epithelial cell line. This was not accompanied by a drastic reduction in mtDNA copy numbers in these cells nor with a reduction in Tfam levels (as we also observed in our study) [73]. It remains to be determined whether PBECs exposed to CS in our study released (damaged) mitochondria through extracellular vesicles. Furthermore, exposure to aldehydes only had a minimal impact on mtDNA abundance and the expression of (subunits of) oxidative phosphorylation complexes. However, aldehyde-induced disruption of mitochondrial function has been reported in the literature, specifically in response to acetaldehyde in hepatocytes [78], to acrolein in cells of the airways, rat liver mitochondria, or rat lung [70,71,75,79,80], to formaldehyde and acetaldehyde in rat liver mitochondria [81], and formaldehyde in neuroblastoma cells [82]. Study of the role of aldehydes in differentiated human PBEC vs. individual aldehydes in non-primary human lung cells may clarify these contrasting results of our study against those of previous studies.

Several in vitro studies using primary airway epithelial cells or cell lines have shown that CS(E) impacts epithelial barrier integrity, induces cilia toxicity, increases mucus production, and changes the presence of cell types in the airway epithelium [83,84,85,86,87,88,89]. This is in line with pathological alterations observed in COPD patients [90,91,92,93]. The absence of changes in mRNA expression of cell-type specific markers in our study may be explained by the acute exposure protocol or by the fact that we used fully differentiated cultures, as studies reporting such changes have often exposed cells during differentiation and/or using repeated or chronic exposure protocols of CS(E) or acrolein [75,76,77,78,79,80,81,82,83,89,94].

**Figure 10:**
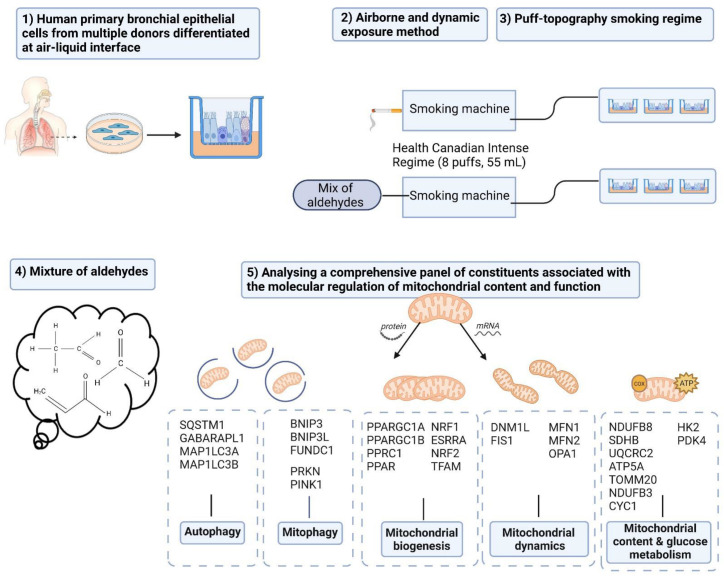
Schematic representation of the strengths and novelties of the study. (1) Use of human primary bronchial epithelial cells from multiple donors differentiated at the air–liquid interface. (2) The airborne and dynamic exposure method and (3) the smoking regime mimicking the puff topography are both more reflective of the in vivo smoking situation, due to the representative chemical characteristics (gaseous and particulate components) of the exposure. (4) Studying the impact of simultaneous exposure to three short-chain aldehydes. (5) Assessment of a comprehensive panel of constituents involved in molecular mechanisms associated with mitochondrial content and function, using our sophisticated in vitro exposure model. Designed with Biorender.com.

Although some of the individual findings that we describe have already been reported in previous studies using other (cell line) models of CS or acrolein exposure, the novelty of our approach and its results is multifaceted. Its strengths and novelties are outlined in this paragraph and in Figure 10. Firstly, some strengths can be observed in the model. The combination of fully differentiated human PBEC with an ALI exposure system can be considered a strength. This model appropriately mimics the human situation, in contrast with studies using submerged cultures of undifferentiated PBEC or immortalized cell lines. Secondly, where previous in vitro studies of submerged cultures have used liquid-based exposure modalities (CSE or aldehydes in liquid formulation), our airborne dynamic exposure method is more reflective of the in vivo situation due to the representative chemical characteristics (gaseous and particulate components). Thirdly, we carefully considered the smoking regime and aimed to mimic the puff topography (i.e., toxic peak concentrations of aldehydes) of smokers in our in vitro model, to study the impact on airway epithelial cells of the mainstream gaseous and particulate components of CS or the mixture of aldehydes. Previous in vitro and in vivo studies mainly used continuous whole CS or even liquid-based exposure. The fourth novelty is that in this experiment we studied for the first time the impact of simultaneous exposure to three short-chain aldehydes. The scarce studies available focused primarily on the impact of individual aldehydes, in particular acrolein [70]. Studying this combination of three aldehydes is important due to indications (based on their common mechanisms and previous co-exposure studies) of an additive or synergistic toxicity [95,96,97,98]. With regard to the data that we obtained, the main novelty is the investigation of a comprehensive panel of essential constituents involved in several molecular mechanisms associated with mitochondrial content and function (mitophagy, mitochondrial dynamics, and mitochondrial biogenesis) and comparison of changes which have never been described before in response to CS and aldehydes.

Obviously, a limitation of our study is that realistic in vitro lung models should also include other cell types in addition to epithelial cells, e.g., macrophages or fibroblasts. Previous studies have shown the potential of such complex co-culture models [99,100,101]. With respect to the dosimetry, we were able to reliably assess the levels of the three aldehydes in the smoke of one cigarette, using a standard operating procedure [55], and the concentrations were in line with previously reported levels [11]. Unfortunately, we were unable to measure levels of the aldehydes in the aldehyde mixture at the site of the cells. However, we have confidence that we generated an aldehyde mixture comparable to the concentrations of acetaldehyde, acrolein, and formaldehyde present in the smoke of one Marlboro Red cigarette. This confidence is based on our calculations using the density and purity of each aldehyde in combination with the total flow of the test atmosphere, as well as the TCA analyses of the aldehyde mixture to check whether each compound was present in the generation of the test atmosphere. However, it should be noted as limitation of our experimental set-up that a mixture of pure aldehydes as used in our study will behave differently from the same quantity of aldehydes present in a mixture with other chemicals, such as in CS. For example, condensation of aldehydes on aerosol particles is more likely in CS. Moreover, as we are interested in the chronic exposure of smoking-associated aldehydes (suggested to be related to COPD pathogenesis), an obvious limitation of our study was the single exposure regime. Nevertheless, acute exposure seems to be partly reflective of changes after chronic exposure, as supported by previous evidence. To briefly illustrate this, effects of short-term CS exposure in our study, especially for the observed induction of autophagy, were in line with observations in lung tissue or PBEC from COPD patients with a long history of smoking [31,33,38,40]. This indicates that our short-term CS exposure model at least partly reflects changes in airway epithelial cells of chronic smokers. In addition, in line with our findings, airway epithelial cells from mice exposed to CS for a longer period of time also showed elevated autophagy markers [102] and upregulated mitophagy [33]. Furthermore, CS exposure for 6 months disrupted mitochondrial metabolism in BEAS-2B cells [31]. Interestingly, Malinska et al. compared short- and long-term CS exposure periods (1 week vs. 12 weeks of CS exposure) in BEAS-2B cells, and found that most changes in mitochondrial function were similar over these two periods, although indications of adaptation were reported after long-term exposure [32].

These limitations should be taken into account in future research, to investigate the relevant gaps in the knowledge. Additional scientific research into the mechanistic and causal involvement of smoking-associated aldehydes in mitochondrial (dys)function in cells of the airways could be of value for supporting regulation of aldehydes in cigarettes. Moreover, further research is required to provide greater insight into the potential link between aldehydes, mitochondrial dysfunction, and COPD pathogenesis, to shed light on potential therapeutical applications targeting aldehydes and/or mitochondria.

Overall, in contrast to our hypothesis, we found no profound impact of aldehydes on molecular mechanisms controlling mitochondrial content and function, compared with CS. This is in contrast to in vitro studies which showed that acrolein or co-exposure to acrolein and formaldehyde disrupts processes involved in the regulation of mitochondrial homeostasis in lung cells [70,79,95,98,103], and conflicts with an in vivo study which observed that proper function of aldehyde dehydrogenase protected against the development of CS-induced airway disease [48]. Probably, the use of our sophisticated and novel exposure model, giving consideration to the above-mentioned corresponding limitations, e.g., dosimetry, could clarify the limited impact of aldehydes on the molecular regulation of pathways involved in mitochondrial content and function. In addition to these study-specific limitations, several other explanations for the absence of a response to aldehydes could be conceived. Theoretically, the combination of the three aldehydes of interest together with any of the other 6000 chemicals present in CS, including other aldehydes, could introduce an additive or synergistic toxicity, which we did not address in our study. For example, a recent study reported enhanced lung carcinogenicity by coexposure to tobacco compound Nitrosamine 4-Methylnitrosamino-1-(3-pyridyl)-1-butanone combined with acetaldehyde, formaldehyde, or carbon dioxide in mice [104]. Moreover, compounds other than these three aldehydes (e.g., carbon monoxide, nicotine, polycyclic aromatic hydrocarbons) may potentially contribute to the mitotoxicity of CS.

In conclusion, this study shows that in vitro exposure of differentiated PBEC to CS disrupts the molecular regulation of mitochondrial content and mitochondrial quality control, while only some of these changes were replicated in response to exposure to a representative combination of aldehydes. These findings suggest that compounds other than aldehydes in CS contribute to CS-induced disruption of the regulation of mitochondrial content and function in airway epithelial cells. Although there is an indication that aldehydes have an impact on molecular mechanisms controlling mitochondrial content and function, additional research should be conducted to provide scientific support for the regulation of aldehydes or other chemicals in CS.

## Figures and Tables

**Table 1:** Characteristics of primary human bronchial epithelial cell donors.

Experiment	Primary Bronchial Epithelial Cell Donors
N	4
Male/female	3/1
Age (years)	69.75 ± 2.02
Body mass index	28.00 ± 4.38
Pack years (years)	35.00 ± 15.00 ^$^
FEV_1_ absolute (L)	2.73 ± 0.19
Tiffeneau Index	76.06 ± 2.06

Subject characteristics of human primary bronchial epithelial cell donors. Data are presented as mean ± s.e.m. ^$^ Missing values for two donors. FEV_1_: forced expiratory volume in first second.

## Data Availability

Not applicable.

## Supplementary Materials

The following are available online at https://www.mdpi.com/article/10.3390/cells11213481/s1, Figure S1: Schematic representation of CS and aldehydes exposure system; Figure S2: Monitoring of the aldehydes present in the mixture using a total carbon analyzer; Figure S3: Validation and characterization of differentiation of PBEC; Figure S4: Monolayer integrity of PBEC during differentiation; Figure S5: No impact of CS or aldehydes exposure on cytotoxicity and monolayer integrity; Figure S6: Exposure to CS or aldehydes resulted in decreased gene expression of *PINK1*; Figure S7: Minor alterations in protein levels of subunits of oxidative phosphorylation complexes following CS or alde-hyde exposure; Figure S8: Minimal changes in mRNA expression of subunits of oxidative phosphorylation complexes upon smoking-associated aldehydes exposure; Figure S9: Bead motion induced by ciliary beating in response to CS or aldehydes exposure; Table S1: Dosimetry Concentration analysis of acetaldehyde, acrolein and formaldehyde in the mainstream smoke of one Marlboro Red cigarette (CS) to match the target concentration of the aldehydes present in the gaseous mixture; Table S2: Human primers sequences used for real time quantitative PCR analysis. Table S3: Target-specific antibodies used for immunohistochemistry staining. Table S4: Antibodies used for western blotting.

## References

[1] GBD 2015 Tobacco Collaborators (2017). Smoking prevalence and attributable disease burden in 195 countries and territories, 1990–2015: A systematic analysis from the Global Burden of Disease Study 2015. Lancet.

[2] Celli B.R., Wedzicha J.A. (2019). Update on Clinical Aspects of Chronic Obstructive Pulmonary Disease. N. Engl. J. Med..

[3] Rodgman A., Perfetti T.A. (2013). The Chemical Components of Tobacco and Tobacco Smoke.

[4] Burns D.M., Dybing E., Gray N., Hecht S., Anderson C., Sanner T., O’Connor R., Djordjevic M., Dresler C., Hainaut P. (2008). Mandated lowering of toxicants in cigarette smoke: A description of the World Health Organization TobReg proposal. Tob. Control.

[5] Cheah N.P. (2016). Volatile Aldehydes in Tobacco Smoke: Source Fate and Risk. Ph.D. Thesis.

[6] Corley R.A., Kabilan S., Kuprat A.P., Carson J.P., Jacob R.E., Minard K.R., Teeguarden J.G., Timchalk C., Pipavath S., Glenny R. (2015). Comparative Risks of Aldehyde Constituents in Cigarette Smoke Using Transient Computational Fluid Dynamics/Physiologically Based Pharmacokinetic Models of the Rat and Human Respiratory Tracts. Toxicol. Sci..

[7] Bos P.M.J., Soeteman-Hernández L.G., Talhout R. (2021). Risk assessment of components in tobacco smoke and e-cigarette aerosols: A pragmatic choice of dose metrics. Inhal. Toxicol..

[8] Yeager R.P., Kushman M., Chemerynski S., Weil R., Fu X., White M., Callahan-Lyon P., Rosenfeldt H. (2016). Proposed Mode of Action for Acrolein Respiratory Toxicity Associated with Inhaled Tobacco Smoke. Toxicol. Sci..

[9] Bernardini L., Barbosa E., Charão M.F., Brucker N. (2020). Formaldehyde toxicity reports from in vitro and in vivo studies: A review and updated data. Drug Chem. Toxicol..

[10] National Research Council (US) Committee (2009). Emergency and Continuous Exposure Guidance Levels for Selected Submarine Contaminants.

[11] Pauwels C., Klerx W.N.M., Pennings J.L.A., Boots A.W., van Schooten F.J., Opperhuizen A., Talhout R. (2018). Cigarette Filter Ventilation and Smoking Protocol Influence Aldehyde Smoke Yields. Chem. Res. Toxicol..

[12] World Health Organization Framework Convention on Tobacco Control Secretariat (2012). Partial Guidelines for Implementation of Articles 9 and 10—Regulation of the Contents of Tobacco Products and Regulation of Tobacco Product Disclosures.

[13] World Health Organization, World Health Organization Tobacco Free Initiative (2008). The Scientific Basis of Tobacco Product Regulation: Second Report of a WHO Study Group.

[14] Hiemstra P.S., Grootaers G., van der Does A.M., Krul C.A.M., Kooter I.M. (2018). Human lung epithelial cell cultures for analysis of inhaled toxicants: Lessons learned and future directions. Toxicol. Vitro.

[15] Crapo J.D., Barry B.E., Gehr P., Bachofen M., Weibel E.R. (1982). Cell number and cell characteristics of the normal human lung. Am. Rev. Respir. Dis..

[16] Hiemstra P.S., McCray P.B., Bals R. (2015). The innate immune function of airway epithelial cells in inflammatory lung disease. Eur. Respir. J..

[17] Cloonan S.M., Choi A.M. (2016). Mitochondria in lung disease. J. Clin. Investig..

[18] Aghapour M., Remels A.H.V., Pouwels S.D., Bruder D., Hiemstra P.S., Cloonan S.M., Heijink I.H. (2020). Mitochondria: At the crossroads of regulating lung epithelial cell function in chronic obstructive pulmonary disease. Am. J. Physiol. Lung Cell. Mol. Physiol..

[19] MacNee W. (2007). Pathogenesis of Chronic Obstructive Pulmonary Disease. Clin. Chest Med..

[20] Hoffmann R.F., Jonker M.R., Brandenburg S.M., de Bruin H.G., Ten Hacken N.H.T., van Oosterhout A.J.M., Heijink I.H. (2019). Mitochondrial dysfunction increases pro-inflammatory cytokine production and impairs repair and corticosteroid responsiveness in lung epithelium. Sci. Rep..

[21] Patergnani S., Bouhamida E., Leo S., Pinton P., Rimessi A. (2021). Mitochondrial Oxidative Stress and “Mito-Inflammation”: Actors in the Diseases. Biomedicines.

[22] Fritsch L.E., Moore M.E., Sarraf S.A., Pickrell A.M. (2020). Ubiquitin and Receptor-Dependent Mitophagy Pathways and Their Implication in Neurodegeneration. J. Mol. Biol..

[23] Lin J., Handschin C., Spiegelman B.M. (2005). Metabolic control through the PGC-1 family of transcription coactivators. Cell Metab..

[24] Scarpulla R.C. (2011). Metabolic control of mitochondrial biogenesis through the PGC-1 family regulatory network. Biochim. Biophys. Acta.

[25] Mishra P., Chan D.C. (2014). Mitochondrial dynamics and inheritance during cell division, development and disease. Nat. Rev. Mol. Cell Biol..

[26] Hara H., Kuwano K., Araya J. (2018). Mitochondrial Quality Control in COPD and IPF. Cells.

[27] Ryter S.W., Rosas I.O., Owen C.A., Martinez F.J., Choi M.E., Lee C.G., Elias J.A., Choi A.M.K. (2018). Mitochondrial Dysfunction as a Pathogenic Mediator of Chronic Obstructive Pulmonary Disease and Idiopathic Pulmonary Fibrosis. Ann. Am. Thorac. Soc..

[28] Agarwal A.R., Yin F., Cadenas E. (2014). Short-term cigarette smoke exposure leads to metabolic alterations in lung alveolar cells. Am. J. Respir. Cell Mol. Biol..

[29] Cloonan S.M., Glass K., Laucho-Contreras M.E., Bhashyam A.R., Cervo M., Pabon M.A., Konrad C., Polverino F., Siempos I.I., Perez E. (2016). Mitochondrial iron chelation ameliorates cigarette smoke-induced bronchitis and emphysema in mice. Nat. Med..

[30] Hara H., Araya J., Ito S., Kobayashi K., Takasaka N., Yoshii Y., Wakui H., Kojima J., Shimizu K., Numata T. (2013). Mitochondrial fragmentation in cigarette smoke-induced bronchial epithelial cell senescence. Am. J. Physiol. Lung Cell. Mol. Physiol..

[31] Hoffmann R.F., Zarrintan S., Brandenburg S.M., Kol A., de Bruin H.G., Jafari S., Dijk F., Kalicharan D., Kelders M., Gosker H.R. (2013). Prolonged cigarette smoke exposure alters mitochondrial structure and function in airway epithelial cells. Respir. Res..

[32] Malinska D., Szymanski J., Patalas-Krawczyk P., Michalska B., Wojtala A., Prill M., Partyka M., Drabik K., Walczak J., Sewer A. (2018). Assessment of mitochondrial function following short- and long-term exposure of human bronchial epithelial cells to total particulate matter from a candidate modified-risk tobacco product and reference cigarettes. Food Chem. Toxicol..

[33] Mizumura K., Cloonan S.M., Nakahira K., Bhashyam A.R., Cervo M., Kitada T., Glass K., Owen C.A., Mahmood A., Washko G.R. (2014). Mitophagy-dependent necroptosis contributes to the pathogenesis of COPD. J. Clin. Investig..

[34] Sundar I.K., Maremanda K.P., Rahman I. (2019). Mitochondrial dysfunction is associated with Miro1 reduction in lung epithelial cells by cigarette smoke. Toxicol. Lett..

[35] Valdivieso Á.G., Dugour A.V., Sotomayor V., Clauzure M., Figueroa J.M., Santa-Coloma T.A. (2018). N-acetyl cysteine reverts the proinflammatory state induced by cigarette smoke extract in lung Calu-3 cells. Redox Biol..

[36] Van der Toorn M., Rezayat D., Kauffman H.F., Bakker S.J., Gans R.O., Koëter G.H., Choi A.M., van Oosterhout A.J., Slebos D.J. (2009). Lipid-soluble components in cigarette smoke induce mitochondrial production of reactive oxygen species in lung epithelial cells. Am. J. Physiol. Lung Cell. Mol. Physiol..

[37] Wu K., Luan G., Xu Y., Shen S., Qian S., Zhu Z., Zhang X., Yin S., Ye J. (2020). Cigarette smoke extract increases mitochondrial membrane permeability through activation of adenine nucleotide translocator (ANT) in lung epithelial cells. Biochem. Biophys. Res. Commun..

[38] Ahmad T., Sundar I.K., Lerner C.A., Gerloff J., Tormos A.M., Yao H., Rahman I. (2015). Impaired mitophagy leads to cigarette smoke stress-induced cellular senescence: Implications for chronic obstructive pulmonary disease. FASEB J..

[39] Chen Z.H., Kim H.P., Sciurba F.C., Lee S.J., Feghali-Bostwick C., Stolz D.B., Dhir R., Landreneau R.J., Schuchert M.J., Yousem S.A. (2008). Egr-1 regulates autophagy in cigarette smoke-induced chronic obstructive pulmonary disease. PLoS ONE.

[40] Ito S., Araya J., Kurita Y., Kobayashi K., Takasaka N., Yoshida M., Hara H., Minagawa S., Wakui H., Fujii S. (2015). PARK2-mediated mitophagy is involved in regulation of HBEC senescence in COPD pathogenesis. Autophagy.

[41] Kyung S.Y., Kim Y.J., Son E.S., Jeong S.H., Park J.W. (2018). The Phosphodiesterase 4 Inhibitor Roflumilast Protects against Cigarette Smoke Extract-Induced Mitophagy-Dependent Cell Death in Epithelial Cells. Tuberc. Respir. Dis..

[42] Mizumura K., Justice M.J., Schweitzer K.S., Krishnan S., Bronova I., Berdyshev E.V., Hubbard W.C., Pewzner-Jung Y., Futerman A.H., Choi A.M.K. (2018). Sphingolipid regulation of lung epithelial cell mitophagy and necroptosis during cigarette smoke exposure. FASEB J..

[43] Park E.J., Park Y.J., Lee S.J., Lee K., Yoon C. (2019). Whole cigarette smoke condensates induce ferroptosis in human bronchial epithelial cells. Toxicol. Lett..

[44] Son E.S., Kim S.H., Ryter S.W., Yeo E.J., Kyung S.Y., Kim Y.J., Jeong S.H., Lee C.S., Park J.W. (2018). Quercetogetin protects against cigarette smoke extract-induced apoptosis in epithelial cells by inhibiting mitophagy. Toxicol. Vitro.

[45] Song C., Luo B., Gong L. (2017). Resveratrol reduces the apoptosis induced by cigarette smoke extract by upregulating MFN2. PLoS ONE.

[46] Zhang M., Shi R., Zhang Y., Shan H., Zhang Q., Yang X., Li Y., Zhang J. (2019). Nix/BNIP3L-dependent mitophagy accounts for airway epithelial cell injury induced by cigarette smoke. J. Cell Physiol..

[47] Wang S., Song X., Wei L., Liu Q., Li C., Wang J. (2022). Role of mitophagy in cigarette smoke-induced lung epithelial cell injury in vitro. Curr. Mol. Med..

[48] Morita K., Masuda N., Oniki K., Saruwatari J., Kajiwara A., Otake K., Ogata Y., Nakagawa K. (2015). Association between the aldehyde dehydrogenase 2*2 allele and smoking-related chronic airway obstruction in a Japanese general population: A pilot study. Toxicol. Lett..

[49] Costa D.L., Kutzman R.S., Lehmann J.R., Drew R.T. (1986). Altered lung function and structure in the rat after subchronic exposure to acrolein. Am. Rev. Respir. Dis..

[50] Tulen C.B.M., Wang Y., Beentjes D., Jessen P.J.J., Ninaber D.K., Reynaert N.L., van Schooten F.J., Opperhuizen A., Hiemstra P.S., Remels A.H.V. (2022). Dysregulated mitochondrial metabolism upon cigarette smoke exposure in various human bronchial epithelial cell models. Dis. Models Mech..

[51] Van der Does A.M., Mahbub R.M., Ninaber D.K., Rathnayake S.N.H., Timens W., van den Berge M., Aliee H., Theis F.J., Nawijn M.C., Hiemstra P.S. (2022). Early transcriptional responses of bronchial epithelial cells to whole cigarette smoke mirror those of in-vivo exposed human bronchial mucosa. Respir. Res..

[52] Van Wetering S., van der Linden A.C., van Sterkenburg M.A., de Boer W.I., Kuijpers A.L., Schalkwijk J., Hiemstra P.S. (2000). Regulation of SLPI and elafin release from bronchial epithelial cells by neutrophil defensins. Am. J. Physiol. Lung Cell. Mol. Physiol..

[53] Van Wetering S., Zuyderduyn S., Ninaber D.K., van Sterkenburg M.A.J.A., Rabe K.F., Hiemstra P.S. (2007). Epithelial differentiation is a determinant in the production of eotaxin-2 and -3 by bronchial epithelial cells in response to IL-4 and IL-13. Mol. Immunol..

[54] World Health Organization, TobLabNet (2012). SOP 1—Standard Operating Procedure for Intense Smoking of Cigarettes.

[55] World Health Organization, TobLabNet (2018). SOP 8—Standard Operating Procedure for Determination of Aldehydes in Mainstream Cigarette Smoke under ISO and Intense Smoking Conditions.

[56] Rasband W.S. (2019). ImageJ, 1997–2018.

[57] Meijering E., Dzyubachyk O., Smal I. (2012). Methods for cell and particle tracking. Methods Enzymol..

[58] Mio T., Romberger D.J., Thompson A.B., Robbins R.A., Heires A., Rennard S.I. (1997). Cigarette smoke induces interleukin-8 release from human bronchial epithelial cells. Am. J. Respir. Crit. Care Med..

[59] Moretto N., Facchinetti F., Southworth T., Civelli M., Singh D., Patacchini R. (2009). alpha, beta-Unsaturated aldehydes contained in cigarette smoke elicit IL-8 release in pulmonary cells through mitogen-activated protein kinases. Am. J. Physiol. Lung Cell. Mol. Physiol..

[60] Dwivedi A.M., Upadhyay S., Johanson G., Ernstgard L., Palmberg L. (2018). Inflammatory effects of acrolein, crotonaldehyde and hexanal vapors on human primary bronchial epithelial cells cultured at air-liquid interface. Toxicol. Vitro.

[61] Nyunoya T., Mebratu Y., Contreras A., Delgado M., Chand H.S., Tesfaigzi Y. (2014). Molecular processes that drive cigarette smoke-induced epithelial cell fate of the lung. Am. J. Respir. Cell Mol. Biol..

[62] Xu W., Janocha A.J., Leahy R.A., Klatte R., Dudzinski D., Mavrakis L.A., Comhair S.A., Lauer M.E., Cotton C.U., Erzurum S.C. (2014). A novel method for pulmonary research: Assessment of bioenergetic function at the air-liquid interface. Redox Biol..

[63] Mavin E., Verdon B., Carrie S., Saint-Criq V., Powell J., Kuttruff C.A., Ward C., Garnett J.P., Miwa S. (2020). Real-time measurement of cellular bioenergetics in fully differentiated human nasal epithelial cells grown at air-liquid-interface. Am. J. Physiol. Lung Cell. Mol. Physiol..

[64] Mizumura K., Cloonan S., Choi M.E., Hashimoto S., Nakahira K., Ryter S.W., Choi A.M. (2016). Autophagy: Friend or Foe in Lung Disease?. Ann. Am. Thorac. Soc..

[65] Jiang S., Sun J., Mohammadtursun N., Hu Z., Li Q., Zhao Z., Zhang H., Dong J. (2019). Dual role of autophagy/mitophagy in chronic obstructive pulmonary disease. Pulm. Pharmacol. Ther..

[66] Monick M.M., Powers L.S., Walters K., Lovan N., Zhang M., Gerke A., Hansdottir S., Hunninghake G.W. (2010). Identification of an autophagy defect in smokers’ alveolar macrophages. J. Immunol..

[67] Fujii S., Hara H., Araya J., Takasaka N., Kojima J., Ito S., Minagawa S., Yumino Y., Ishikawa T., Numata T. (2012). Insufficient autophagy promotes bronchial epithelial cell senescence in chronic obstructive pulmonary disease. Oncoimmunology.

[68] Barnes P.J., Baker J., Donnelly L.E. (2022). Autophagy in asthma and chronic obstructive pulmonary disease. Clin. Sci..

[69] Leonard A.P., Cameron R.B., Speiser J.L., Wolf B.J., Peterson Y.K., Schnellmann R.G., Beeson C.C., Rohrer B. (2015). Quantitative analysis of mitochondrial morphology and membrane potential in living cells using high-content imaging, machine learning, and morphological binning. Biochim. Biophys. Acta.

[70] Wang H.T., Lin J.H., Yang C.H., Haung C.H., Weng C.W., Maan-Yuh Lin A., Lo Y.L., Chen W.S., Tang M.S. (2017). Acrolein induces mtDNA damages, mitochondrial fission and mitophagy in human lung cells. Oncotarget.

[71] Tulen C.B.M., Snow S.J., Leermakers P.A., Kodavanti U.P., van Schooten F.J., Opperhuizen A., Remels A.H.V. (2022). Acrolein inhalation acutely affects the regulation of mitochondrial metabolism in rat lung. Toxicology.

[72] Liu Q.P., Zhou D.X., Lv M.Q., Ge P., Li Y.X., Wang S.J. (2018). Formaldehyde inhalation triggers autophagy in rat lung tissues. Toxicol. Ind. Health.

[73] Giordano L., Gregory A.D., Verdaguer M.P., Ware S.A., Harvey H., DeVallance E., Brzoska T., Sundd P., Zhang Y., Sciurba F.C. (2022). Extracellular release of mitochondrial DNA: Triggered by cigaretee smoke and detected in COPD. Cells.

[74] Vanella L., Li Volti G., Distefano A., Raffaele M., Zingales V., Avola R., Tibullo D., Barbagallo I. (2017). A new antioxidant formulation reduces the apoptotic and damaging effect of cigarette smoke extract on human bronchial epithelial cells. Eur. Rev. Med. Pharmacol. Sci..

[75] Luo C., Li Y., Yang L., Feng Z., Li Y., Long J., Liu J. (2013). A cigarette component acrolein induces accelerated senescence in human diploid fibroblast IMR-90 cells. Biogerontology.

[76] Aravamudan B., Kiel A., Freeman M., Delmotte P., Thompson M., Vassallo R., Sieck G.C., Pabelick C.M., Prakash Y.S. (2014). Cigarette smoke-induced mitochondrial fragmentation and dysfunction in human airway smooth muscle. Am. J. Physiol. Lung Cell. Mol. Physiol..

[77] Ballweg K., Mutze K., Königshoff M., Eickelberg O., Meiners S. (2014). Cigarette smoke extract affects mitochondrial function in alveolar epithelial cells. Am. J. Physiol. Lung Cell. Mol. Physiol..

[78] Farfán Labonne B.E., Gutiérrez M., Gómez-Quiroz L.E., Konigsberg Fainstein M., Bucio L., Souza V., Flores O., Ortíz V., Hernández E., Kershenobich D. (2009). Acetaldehyde-induced mitochondrial dysfunction sensitizes hepatocytes to oxidative damage. Cell Biol. Toxicol..

[79] Agarwal A.R., Yin F., Cadenas E. (2013). Metabolic shift in lung alveolar cell mitochondria following acrolein exposure. Am. J. Physiol. Lung Cell. Mol. Physiol..

[80] Sun L., Luo C., Long J., Wei D., Liu J. (2006). Acrolein is a mitochondrial toxin: Effects on respiratory function and enzyme activities in isolated rat liver mitochondria. Mitochondrion.

[81] Van Buskirk J.J., Frisell W.R. (1969). Inhibition by formaldehyde of energy transfer and related processes in rat liver mitochondria. II. Effects on energy-linked reactions in intact mitochondria and phosphorylating particles. Arch. Biochem. Biophys..

[82] Zerin T., Kim J.S., Gil H.W., Song H.Y., Hong S.Y. (2015). Effects of formaldehyde on mitochondrial dysfunction and apoptosis in SK-N-SH neuroblastoma cells. Cell Biol. Toxicol..

[83] Schamberger A.C., Staab-Weijnitz C.A., Mise-Racek N., Eickelberg O. (2015). Cigarette smoke alters primary human bronchial epithelial cell differentiation at the air-liquid interface. Sci. Rep..

[84] Kuehn D., Majeed S., Guedj E., Dulize R., Baumer K., Iskandar A., Boue S., Martin F., Kostadinova R., Mathis C. (2015). Impact assessment of repeated exposure of organotypic 3D bronchial and nasal tissue culture models to whole cigarette smoke. J. Vis. Exp..

[85] Aufderheide M., Scheffler S., Ito S., Ishikawa S., Emura M. (2015). Ciliatoxicity in human primary bronchiolar epithelial cells after repeated exposure at the air–liquid interface with native mainstream smoke of K3R4F cigarettes with and without charcoal filter. Exp. Toxicol. Pathol..

[86] Tatsuta M., Kan-o K., Ishii Y., Yamamoto N., Ogawa T., Fukuyama S., Ogawa A., Fujita A., Nakanishi Y., Matsumoto K. (2019). Effects of cigarette smoke on barrier function and tight junction proteins in the bronchial epithelium: Protective role of cathelicidin LL-37. Respir. Res..

[87] Yu Q., Chen X., Fang X., Chen Q., Hu C. (2015). Caveolin-1 aggravates cigarette smoke extract-induced MUC5AC secretion in human airway epithelial cells. Int. J. Mol. Med..

[88] Brekman A., Walters M.S., Tilley A.E., Crystal R.G. (2014). FOXJ1 prevents cilia growth inhibition by cigarette smoke in human airway epithelium in vitro. Am. J. Respir. Cell Mol. Biol..

[89] Gindele J.A., Kiechle T., Benediktus K., Birk G., Brendel M., Heinemann F., Wohnhaas C.T., LeBlanc M., Zhang H., Strulovici-Barel Y. (2020). Intermittent exposure to whole cigarette smoke alters the differentiation of primary small airway epithelial cells in the air-liquid interface culture. Sci. Rep..

[90] Rigden H.M., Alias A., Havelock T., O’Donnell R., Djukanovic R., Davies D.E., Wilson S.J. (2016). Squamous Metaplasia Is Increased in the Bronchial Epithelium of Smokers with Chronic Obstructive Pulmonary Disease. PLoS ONE.

[91] Crystal R.G. (2014). Airway basal cells. The “smoking gun” of chronic obstructive pulmonary disease. Am. J. Respir. Crit. Care Med..

[92] Jeffery P.K. (2000). Comparison of the structural and inflammatory features of COPD and asthma Giles F. Filley Lecture. Chest.

[93] Saetta M., Turato G., Baraldo S., Zanin A., Braccioni F., Mapp C.E., Maestrelli P., Cavallesco G., Papi A., Fabbri L.M. (2000). Goblet cell hyperplasia and epithelial inflammation in peripheral airways of smokers with both symptoms of chronic bronchitis and chronic airflow limitation. Am. J. Respir. Crit. Care Med..

[94] Xiong R., Wu Q., Muskhelishvili L., Davis K., Shemansky J.M., Bryant M., Rosenfeldt H., Healy S.M., Cao X. (2018). Evaluating Mode of Action of Acrolein Toxicity in an In Vitro Human Airway Tissue Model. Toxicol. Sci..

[95] Zhang S., Zhang J., Chen H., Wang A., Liu Y., Hou H., Hu Q. (2019). Combined cytotoxicity of co-exposure to aldehyde mixtures on human bronchial epithelial BEAS-2B cells. Environ. Pollut..

[96] LoPachin R.M., Gavin T. (2014). Molecular mechanisms of aldehyde toxicity: A chemical perspective. Chem. Res. Toxicol..

[97] Zhang S., Chen H., Wang A., Liu Y., Hou H., Hu Q. (2018). Combined effects of co-exposure to formaldehyde and acrolein mixtures on cytotoxicity and genotoxicity in vitro. Environ. Sci. Pollut. Res. Int..

[98] Zhang S., Zhang J., Cheng W., Chen H., Wang A., Liu Y., Hou H., Hu Q. (2020). Combined cell death of co-exposure to aldehyde mixtures on human bronchial epithelial BEAS-2B cells: Molecular insights into the joint action. Chemosphere.

[99] Iskandar A.R., Xiang Y., Frentzel S., Talikka M., Leroy P., Kuehn D., Guedj E., Martin F., Mathis C., Ivanov N.V. (2015). Impact Assessment of Cigarette Smoke Exposure on Organotypic Bronchial Epithelial Tissue Cultures: A Comparison of Mono-Culture and Coculture Model Containing Fibroblasts. Toxicol. Sci..

[100] Van Riet S., van Schadewijk A., de Vos S., Vandeghinste N., Rottier R.J., Stolk J., Hiemstra P.S., Khedoe P. (2020). Modulation of Airway Epithelial Innate Immunity and Wound Repair by M(GM-CSF) and M(M-CSF) Macrophages. J. Innate Immun..

[101] Ishikawa S., Matsumura K., Kitamura N., Takanami Y., Ito S. (2019). Multi-omics analysis: Repeated exposure of a 3D bronchial tissue culture to whole-cigarette smoke. Toxicol. Vitro.

[102] Chen Z.H., Lam H.C., Jin Y., Kim H.P., Cao J., Lee S.J., Ifedigbo E., Parameswaran H., Ryter S.W., Choi A.M. (2010). Autophagy protein microtubule-associated protein 1 light chain-3B (LC3B) activates extrinsic apoptosis during cigarette smoke-induced emphysema. Proc. Natl. Acad. Sci. USA.

[103] Liu D., Cheng Y., Tang Z., Mei X., Cao X., Liu J. (2022). Toxicity mechanism of acrolein on DNA damage and apoptosis in BEAS-2B cells: Insights from cell biology and molecular docking analyses. Toxicology.

[104] Peterson L.A., Oram M.K., Flavin M., Seabloom D., Smith W.E., O’Sullivan M.G., Vevang K.R., Upadhyaya P., Stornetta A., Floeder A.C. (2021). Coexposure to Inhaled Aldehydes or Carbon Dioxide Enhances the Carcinogenic Properties of the Tobacco-Specific Nitrosamine 4-Methylnitrosamino-1-(3-pyridyl)-1-butanone in the A/J Mouse Lung. Chem. Res. Toxicol..

