# Supplementary material for: Smoking-Associated Exposure of Human Primary Bronchial Epithelial Cells to Aldehydes: Impact on Molecular Mechanisms Controlling Mitochondrial Content and Function
Source: Cells. 2022 Nov 3;11(21):3481. doi: 10.3390/cells11213481 (PMC9655975; doi:10.3390/cells11213481)
Supplement: Supplementary file 1 [file cells-11-03481-s001.zip › cells-1822376-supplementary.pdf]

Supplementary Data

Smoking-Associated Exposure of Human Primary Bronchial Epithelial Cells to Aldehydes: Impact on Molecular Mechanisms Controlling Mitochondrial Content and Function

Christy B. M. Tulen [1], Evert Duistermaat [2], Johannes W. J. M. Cremers [2], Walther N. M. Klerx [2], Paul H. B. Fokkens [2], Naömi Weibolt [2], Nico Kloosterboer [3,4], Mieke A. Dentener [4,5], Eric R. Gremmer [2], Phyllis J. J. Jessen [1], Evi J. C. Koene [1], Lou Maas [1], Antoon Opperhuizen [1,6], Frederik-Jan van Schooten [1], Yvonne C. M. Staal [2] and Alexander H. V. Remels [1,*]

1 School of Nutrition and Translational Research in Metabolism (NUTRIM), Department of Pharmacology and Toxicology, Maastricht University Medical Center+, 6200 MD Maastricht, The Netherlands
2 National Institute for Public Health and the Environment (RIVM), 3721 MA Bilthoven, The Netherlands
3 Department of Pediatrics, Maastricht University Medical Center+, 6229 HX Maastricht, The Netherlands
4 Primary Lung Culture (PLUC) Facility, Maastricht University Medical Center+, 6200 MD Maastricht, The Netherlands
5 School of Nutrition and Translational Research in Metabolism (NUTRIM), Department of Respiratory Medicine, Maastricht University Medical Center+, 6200 MD Maastricht, The Netherlands
6 Office of Risk Assessment and Research, Netherlands Food and Consumer Product Safety Authority (NVWA), 3511 GG Utrecht, The Netherlands
* Correspondence: a.remels@maastrichtuniversity.nl

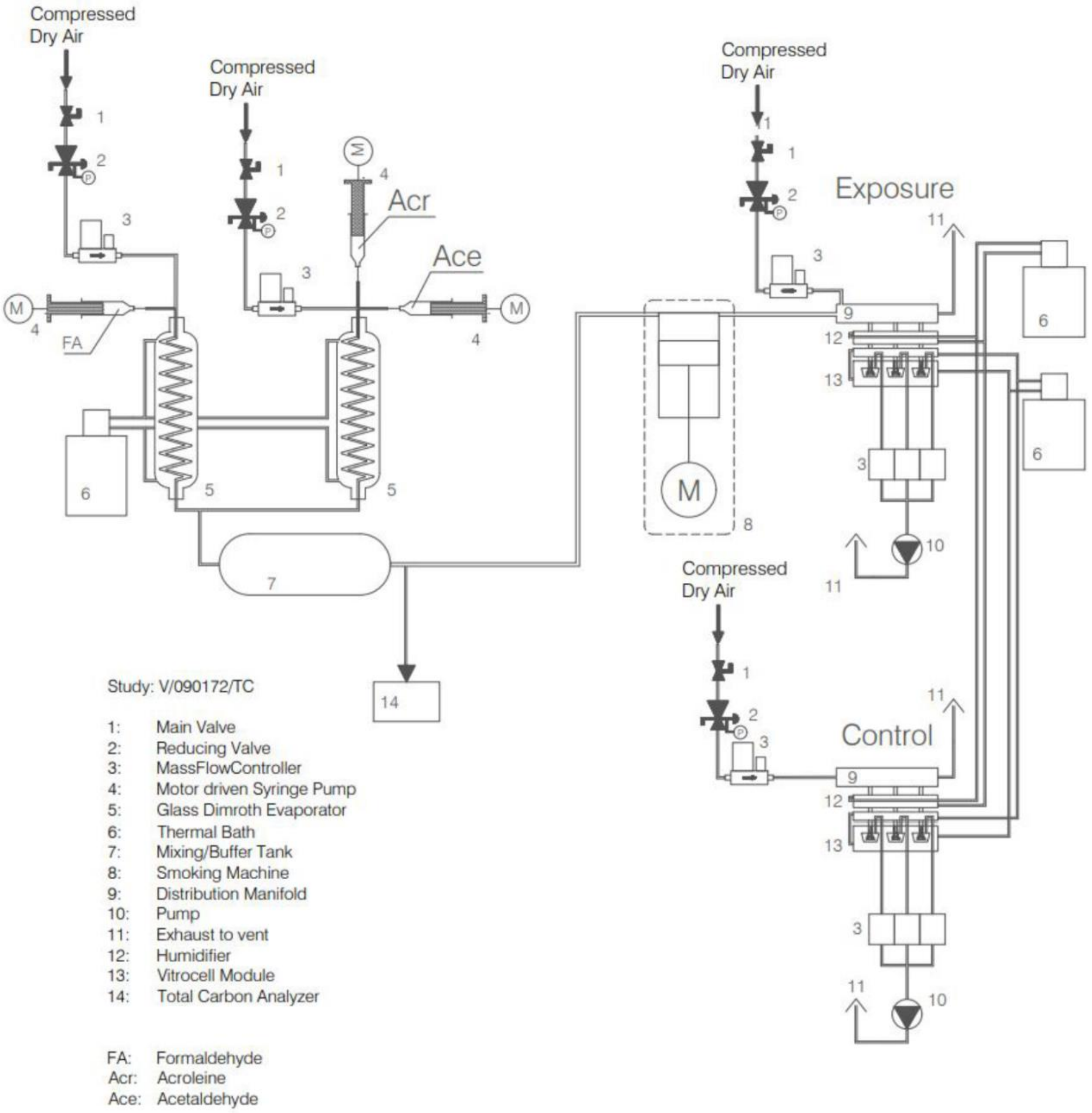

Figure S1. Schematic representation of CS and aldehydes exposure system. Schematic representation of the designed exposure system used to direct expose differentiated PBEC cultures to cigarette smoke (CS) or a comparable dose mixture of aldehydes via the air using two Vitrocell® 12/3 CF stainless steel modules for 12-well sized inserts (Vitrocell, Germany). The mixture of 3 aldehydes was generated by evaporating syringe pump controlled flows of the aldehydes in streams of mass flow controlled compressed dry air using two Dimroth evaporators (one for formaldehyde, the other one for acrolein and acetaldehyde). After evaporation, the generated formaldehyde and acrolein/acetaldehyde streams were mixed in a buffer chamber. A Total Carbon Analyzer (TCA, Ratfisch, Germany) was used to monitor the concentrations of aldehydes during every exposure. PBEC were exposed to 8 puffs of CS, gaseous mixture of aldehydes or compressed dry air following the Health Canada Intense smoking regime. The generated streams of CS or aldehydes were distributed (and diluted) to the exposure manifold by a mass flow controlled stream of 500 mL/min compressed dry air. Subsequently, streams of 5 mL/min per insert were extracted from the exposure manifold. These streams were individually humidified by passing them through Nafion humidifiers kept in a water bath at 33.8 °C. After humidification the streams were delivered to the cells. As control, the PBEC cultures were exposed to clean compressed air with a similar flow-rate and humidification in the control module.

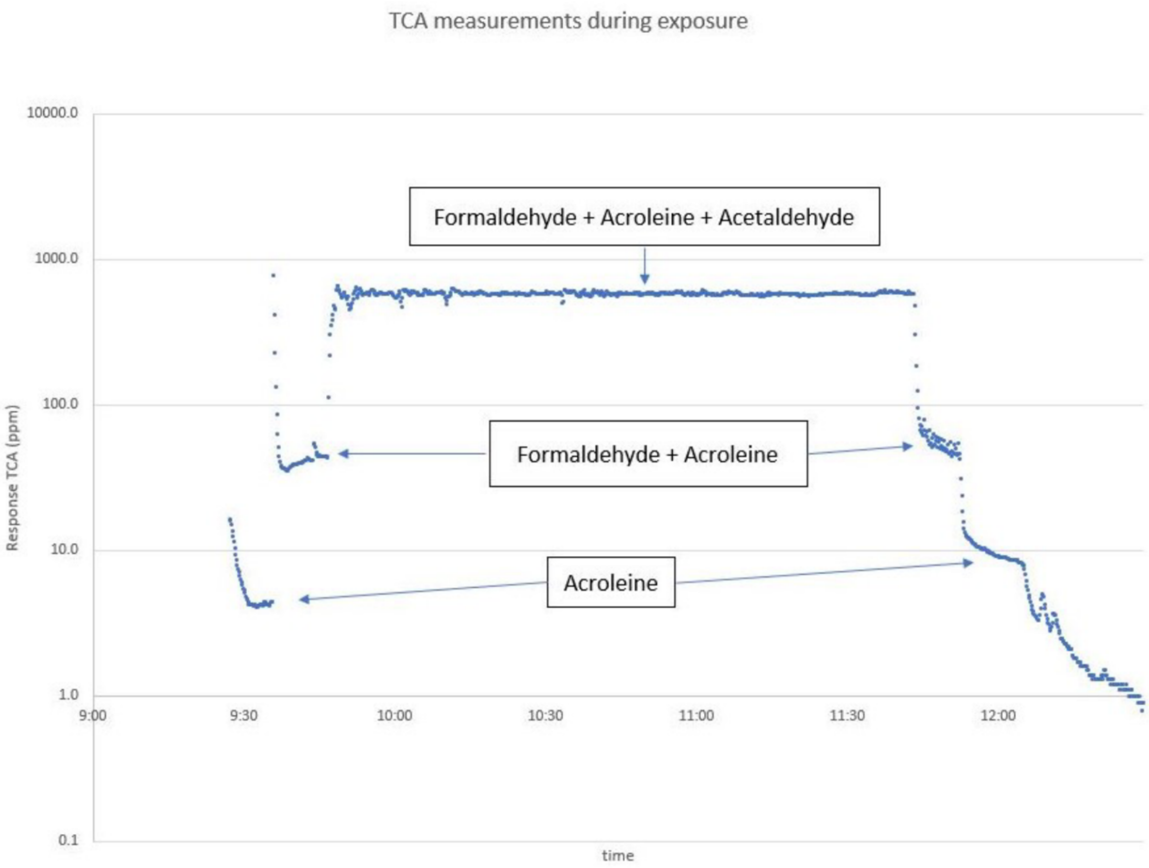

Figure S2. Monitoring of the aldehydes present in the mixture using a total carbon analyzer. A Total Carbon Analyzer (TCA, Ratfisch, Germany) was used to monitor the concentrations of aldehydes during every exposure. The response of the TCA was used as an indicator of the presence of each of the aldehydes in the gaseous mixture (located before the smoking machine). Generation started with formaldehyde (with a slight overshoot in the beginning). After stabilization, the generation of acrolein was started (also with a slight overshoot in the beginning) before initiating the acetaldehyde generation. After the last exposure the generation of acetaldehyde was stopped first, followed by acrolein and, after stabilization, also the generation of formaldehyde was terminated. This example of a sequence of one exposure experiment is showing the attribution of each individual aldehyde to the measured total concentration.

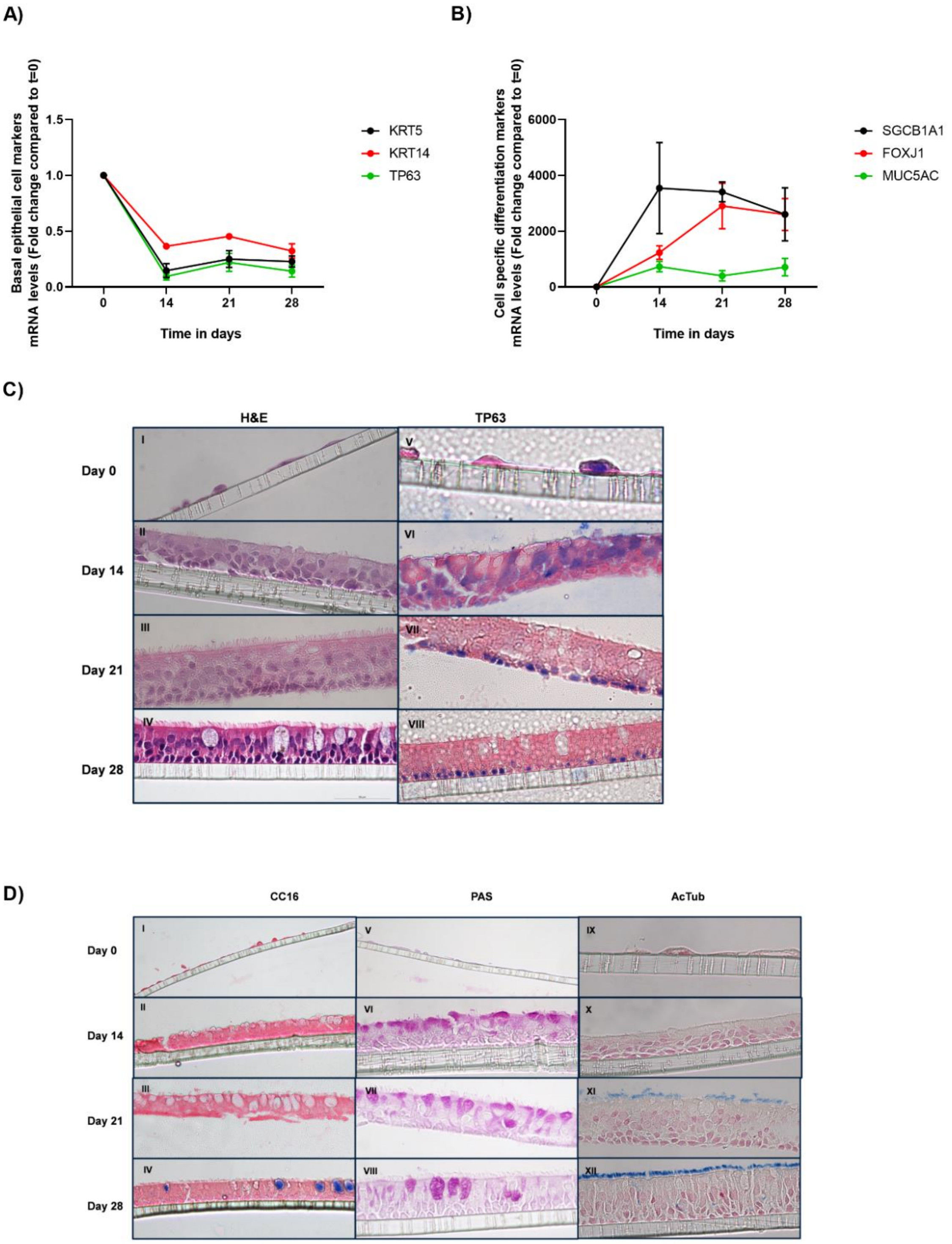

Figure S3. Validation and characterization of differentiation of PBEC. Validation and characterization of differentiation of human primary bronchial epithelial cells (PBEC) monitored at several time-points: day 0 (moment of air-lift) and days 14, 21 and 28 post-airlift. **(A)** mRNA expression of markers associated with basal epithelial cells: *Keratin 5* (*KRT5*; black), *Keratin 14* (*KRT14*; red), and nuclear *Tumor protein 63* (*TP63*; green) are presented. **(B)** Expression of genes representing specific cell types: club cells (*Secretoglobin Familiy 1A Member 1*; *SCGB1A1*) (black), ciliated cells (*Forkhead Box J1*; *FOXJ1*) (red) and goblet cells (*Mucin 5AC, Oligomeric Mucus/Gel-Forming*; *MUC5AC*) (green) are presented. Data are presented as the mean fold change of the two donors (i.e., the mean of biological triplicates per donor were averaged) per time-point compared to day 0 (moment of air-lift) ± s.e.m. Further verification of the differentiation status of PBEC at different time points is conducted by **(C)** a hematoxyline-eosine (H&E) staining (I-IV) and TP63 (nuclei) (V-VIII) as well as by **(D)** immunohistochemistry stainings with target-specific antibodies: CC16 (clara cell protein 16) (I-IV), Periodic acid-Schiff (PAS; polysaccharides and mucosubstances) (V-VIII), and Acetylated Tubulin (cilia) (IX-XII). Microscopy images (magnification 200X or 400X) of one representative donor were shown.

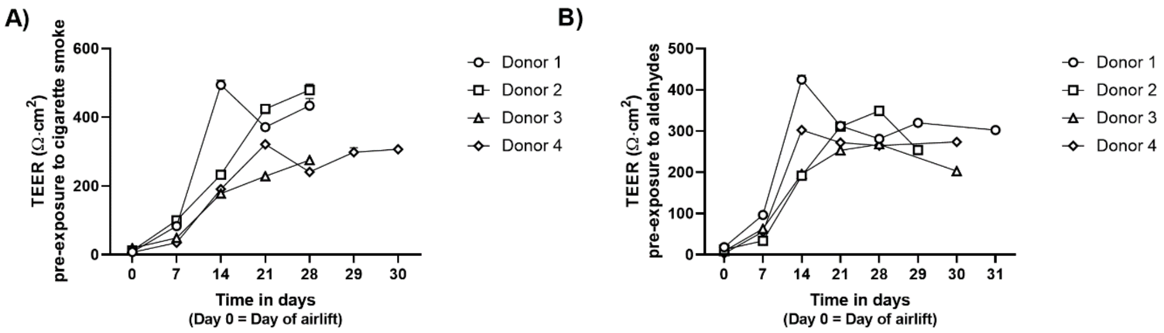

Figure S4. Monolayer integrity of PBEC during differentiation. The monolayer integrity, trans-epithelial electrical resistance (TEER; Ω·cm2) was monitored during differentiation of the PBEC of all donors (n=4) in all inserts before exposure to **(A)** CS and **(B)** aldehydes. Data are presented as mean TEER per donor ± s.e.m. The mean of replicates per independent donor (experiment) (n=9-90) are represented by open circles, triangles, squares or diamonds.

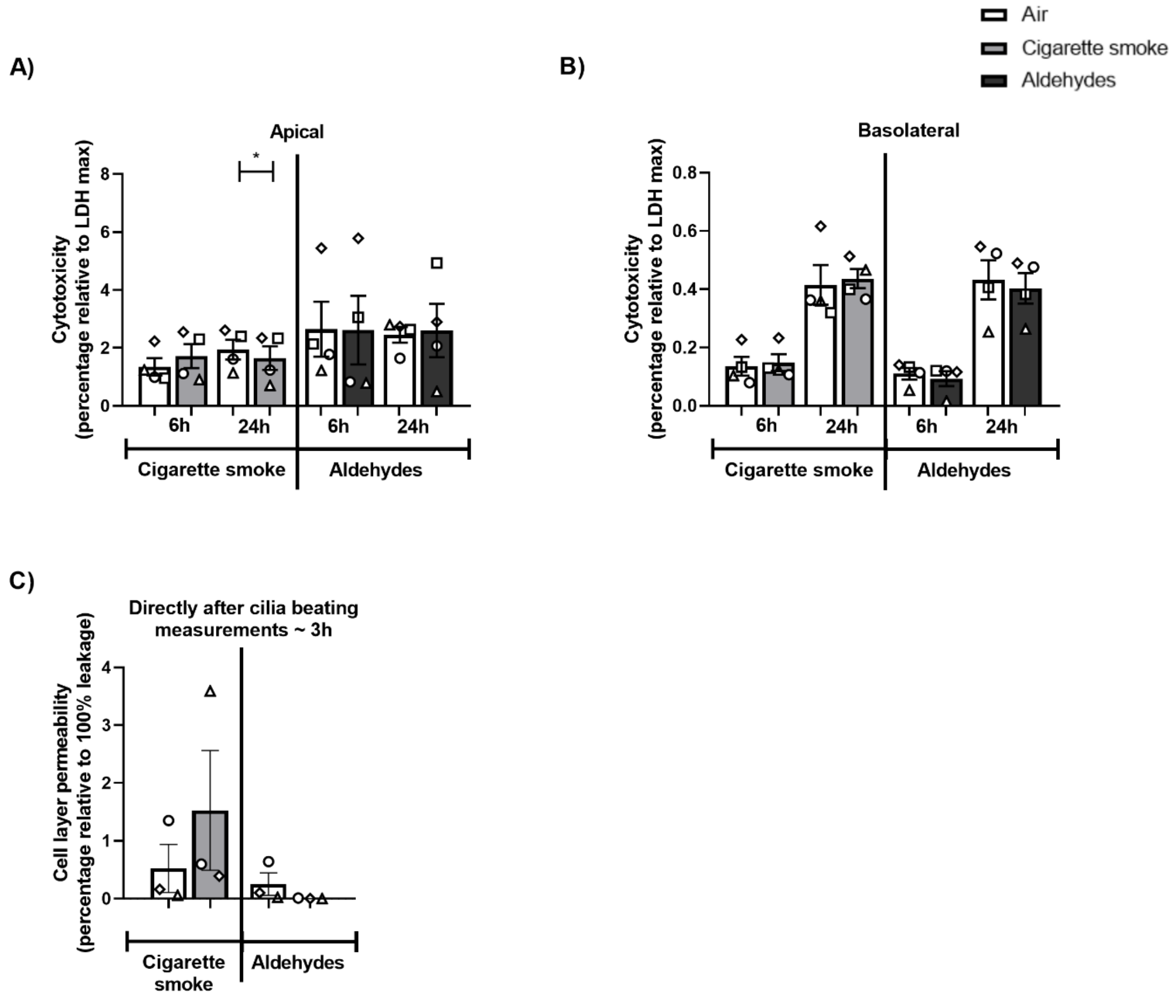

Figure S5. No impact of CS or aldehydes exposure on cytotoxicity and monolayer integrity. Differentiated human primary bronchial epithelial cells (PBEC) from non-COPD subjects were exposed in a continuous flow system using a puff-like exposure protocol to smoke of one Marlboro Red cigarette (CS) or a mixture of aldehydes: acetaldehyde, acrolein, and formaldehyde (at relevant concentrations of 1 cigarette) or air (control) (n=3-4 donors). **(A)** After recovery for 6 or 24 h, cytotoxicity was analysed by the LDH assay (percentage relative to LDH max). **(B,C)** Moreover, following exposure and cilia beating analysis (±3 h of recovery), cell layer permeability was analysed (percentage relative to 100% leakage control). Data are presented as mean fold change compared to relative positive control ± s.e.m. The mean of biological triplicates per independent donor are represented by open circles, triangles, squares or diamonds. Statistical differences between CS *versus* air or a mixture of aldehydes *versus* air were tested using a two-tailed paired parametric t-test. Statistical significance is indicated as $^{*}p<0.05$ *versus* air (control).

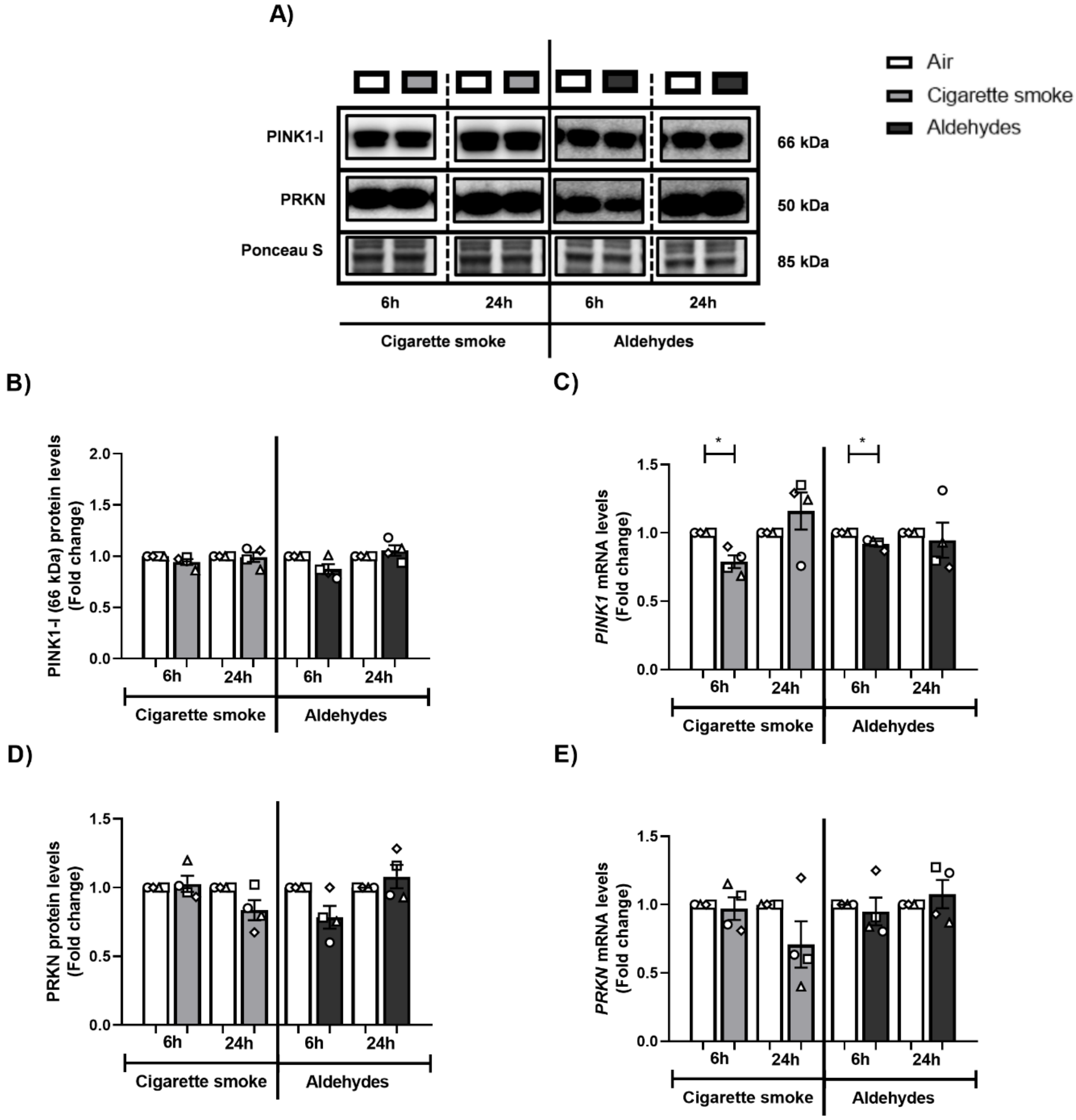

Figure S6. Exposure to CS or aldehydes resulted in decreased gene expression of *PINK1*. Differentiated human primary bronchial epithelial cells (PBEC) from non-COPD subjects were exposed in a continuous flow system using a puff-like exposure protocol to smoke of one Marlboro Red cigarette (CS) or a mixture of aldehydes: acetaldehyde, acrolein, and formaldehyde (at relevant concentrations of 1 cigarette) or air (control) (n=4 donors). Following recovery for 6 or 24 h, whole cell lysates were harvested for analysis of the abundance of key regulators involved in ubiquitin-mediated mitophagy, respectively protein **(A, B, D)** PINK1-I (66 kDa) and PRKN as well as transcript **(C, E)** levels of *PINK1* and *PRKN*. Representative western blot images are shown of one replicate of one donor/experiment reflective of the changes in all donors as quantified in the corresponding graph. Data are presented as mean fold change compared to air control ± s.e.m. The mean of biological triplicates per independent donor are represented by open circles, triangles, squares or diamonds. Statistical differences between CS *versus* air or a mixture of aldehydes *versus* air were tested using a two-tailed paired parametric t-test. Statistical significance is indicated as *$p<0.05$ *versus* air (control).

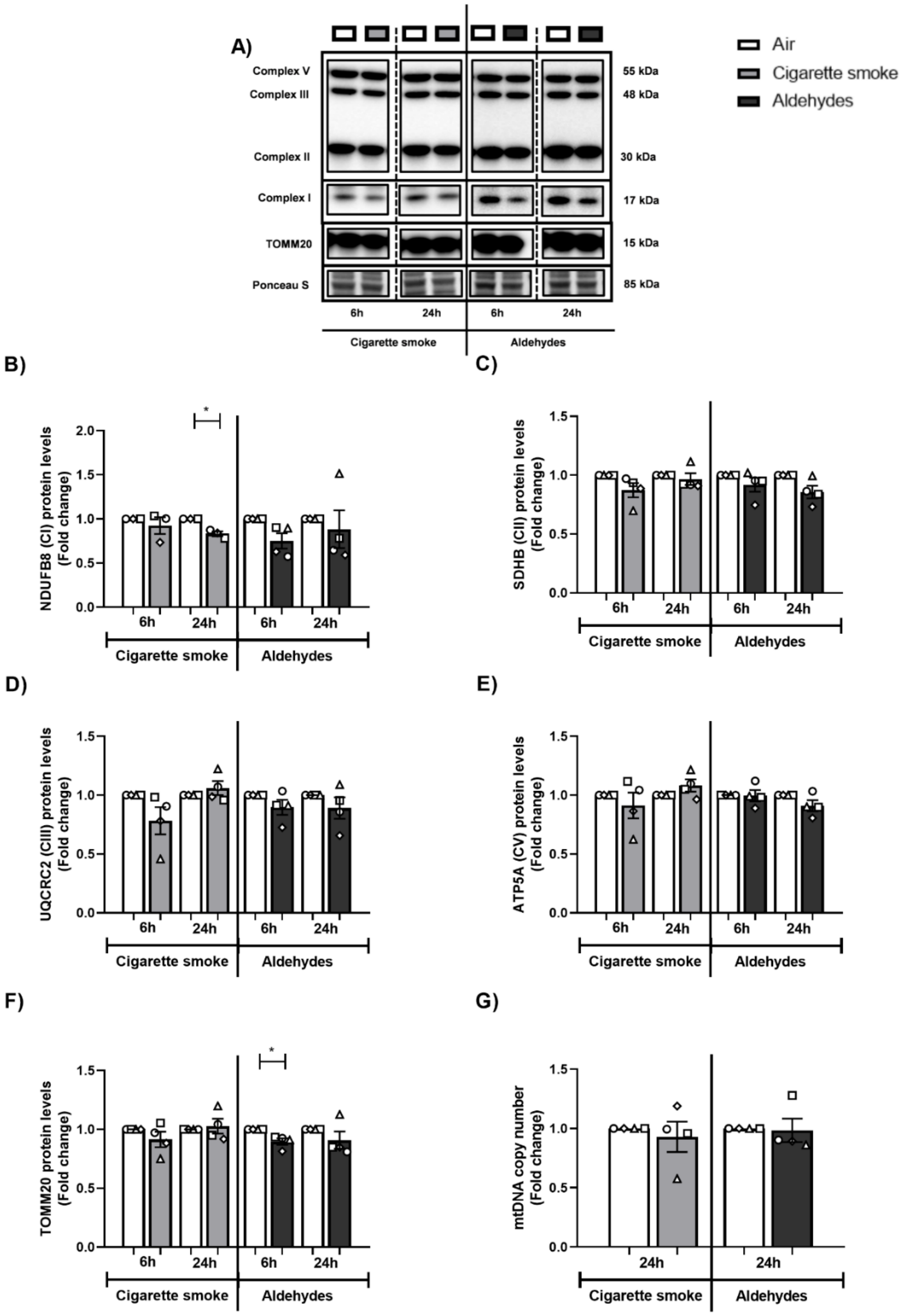

Figure S7. Minor alterations in protein levels of subunits of oxidative phosphorylation complexes following CS or aldehyde exposure. Differentiated human primary bronchial epithelial cells (PBEC) from non-COPD subjects were exposed in a continuous flow system using a puff-like exposure protocol to smoke of one Marlboro Red cigarette (CS) or a mixture of aldehydes: acetaldehyde, acrolein, and formaldehyde (at relevant concentrations of 1 cigarette) or air (control) (n=3-4 donors). After recovery for 6 or 24 h, whole cell lysates were harvested to analyze (**A**) protein levels of subunits of (**B**) NDUFB8 (complex I), (**C**) SDHB (complex II), (**D**) UQCRC2 (complex III), (**E**) ATP5A (complex V), (**F**) TOMM20 and (**G**) mtDNA copy number (*MT-CO2*/*ACTB*). Representative western blot images are shown of one replicate of one donor/ ex-periment reflective of the changes in all donors as quantified in the corresponding graph. Data are presented as mean fold change compared to air control ± s.e.m. The mean of biological triplicates per independent donor are represented by open circles, triangles, squares or diamonds. Statistical differences between CS *versus* air or a mixture of aldehydes *versus* air were tested using a two-tailed paired parametric t-test. Statistical significance is indicated as *p<0.05 *versus* air (control).

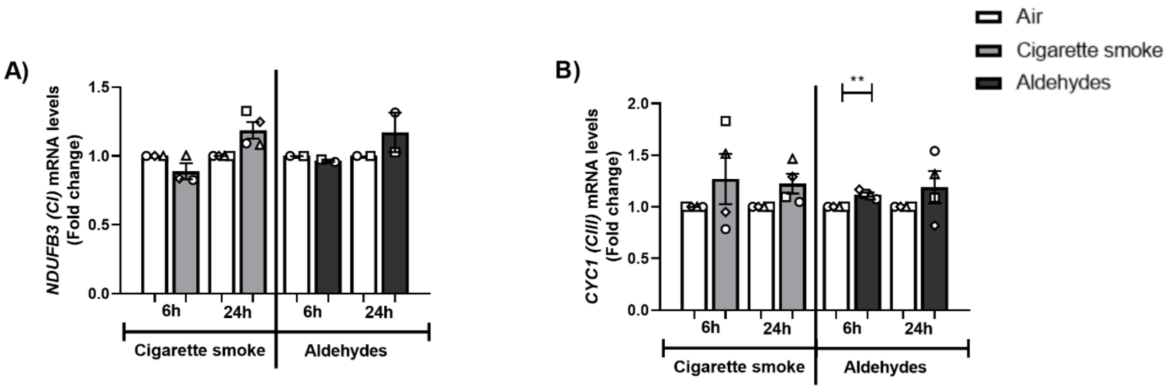

Figure S8. Minimal changes in mRNA expression of subunits of oxidative phosphorylation complexes upon smoking-associated aldehydes exposure. Differentiated human primary bronchial epithelial cells (PBEC) from non-COPD subjects were exposed in a continuous flow system using a puff-like exposure protocol to smoke of one Marlboro Red cigarette (CS) or a mixture of aldehydes: acetaldehyde, acrolein, and formaldehyde (at relevant concentrations of 1 cigarette) or air (control) (n=2-4 donors). After recovery for 6 or 24 h, whole cell lysates were harvested to analyze transcript levels of (**A**) *NDUFB3* (*CI*), and (**B**) *CYC1* (*CIII*). Data are presented as mean fold change compared to air control ± s.e.m. The mean of biological triplicates per independent donor are represented by open circles, triangles, squares or diamonds. Statistical differences between CS *versus* air or a mixture of aldehydes *versus* air were tested using a two-tailed paired parametric t-test. Statistical significance is indicated as **p<0.01 *versus* air (control).

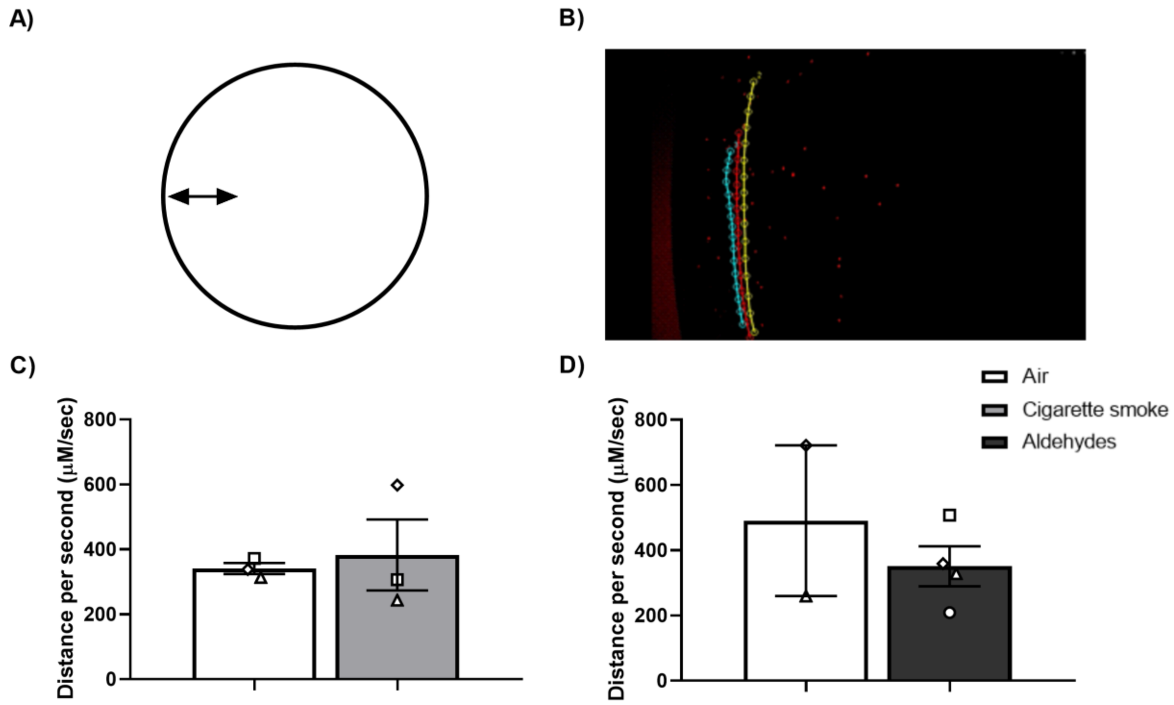

Figure S9. Bead motion induced by ciliary beating in response to CS or aldehydes exposure. Differentiated human primary bronchial epithelial cells (PBEC) from non-COPD subjects were exposed in a continuous flow system using a puff-like exposure protocol to smoke of one Marlboro Red cigarette (CS) or a mixture of aldehydes: acetaldehyde, acrolein, and formaldehyde (at relevant concentrations of 1 cigarette) or air (control) (n=2-4 donors). Immediately after exposure, 2.1 μM beads were added to the apical side of the exposed PBEC in the middle of the transwell. The exposed PBEC were placed on the microscope (Leica Dmi8), and maintained at 37°C and 5% CO_2 in humidified air. Videos of the bead motion were acquired 30 min until 2.5 h post-exposure using the 4x objective and LEICA DFC7000 GT camera along with the LAS X 3.4.2 software, respectively in a frame interval of 0.33 sec for 20-60 sec. **(A)** Beads which were located as close as possible to the edge of the transwell, outside the mucus droplet and following a smooth path were selected for tracking. Automatic tracking of the beads was conducted in ImageJ using the MTrackJ plugin (scale: 0.2065 pixels/μM), **(B)** see an example of tracking of 3 beads of donor 3: CS exposure. Per exposure condition (CS, aldehydes, air), if available, 1 to 3 videos per transwell per donor were selected. Within one video (i.e., one transwell), 5-6 beads were tracked for 2.97-5.00 sec (i.e., 9-16 time frames). Mean distance per sec was calculated per exposure condition per donor ± s.e.m., respectively including tracked beads in 1-3 videos resulting in **(C)** 5-12 automatic tracked beads per donor exposed to air or CS or **(D)** 6-23 tracked beads per donor exposed to air or aldehydes. In summary, this results in a total number of tracked beads across all donors of air: 29 versus CS: 24 and air: 18 versus aldehydes: 41. Means per independent donor are represented by open circles, triangles, squares or diamonds. Statistical differences between CS *versus* air or a mixture of aldehydes *versus* air were tested using a two-tailed paired parametric t-test.

Table S1: Dosimetry Concentration analysis of acetaldehyde, acrolein and formaldehyde in the mainstream smoke of one Marlboro Red cigarette (CS) to match the target concentration of the aldehydes present in the gaseous mixture. Marlboro Red cigarettes (taped) were smoked for 8 puffs following the Health Canada Intense Bell regime (2 puffs/min, 55 ml puff volume, 2 sec puff duration, 4 sec puff exhaust time) using a smoking machine (VC1, Vitrocell, Germany). After priming the system by smoking 3 cigarettes in a row, the mainstream smoke from one cigarette was trapped using a Cambridge filter and Carboxen adsorbent cartridge (extern filter) directly after the smoking machine. Besides the primary trapping cartridge, an additional cartridge was included to measure possible flow through. Measurements were conducted under the following circumstances: 500 mL/min dilution flow; 1 L/min sample flow – continuous, exhaust flow smoking machine complemented with clean compressed air. Data are presented as mean concentration of individual aldehyde (mg/m^3) as measured in the trapped mainstream smoke of 3 individual cigarettes including flow through ± s.d.

**Source**	**Exposure component**	**Acetaldehyde (mg/m^3)**	**Acrolein (mg/m^3)**	**Formaldehyde (mg/m^3)**
**Literature[1]**	**Marlboro Red Cigarette**	2121.4	227.5	105.2
**Study**	**Marlboro Red Cigarette**	2127.1 ± 264.5	137.7 ± 38.3	103.4 ± 15.3

Table S2. Human primers sequences used for real time quantitative PCR analysis.

Gene	Sense primer (5′-3′)	Antisense primer (3′-5′)
**Reference genes**		
ACTB	AAGCCACCCCACTTCTCTCTAA	AATGCTATCACCTCCCCTGTGT
B2M	CTGTGCTCGCGCTACTCTCTCTT	TGAGTAAACCTGAATCTTTGGAGTACGC
PPIA	CATCTGCACTGCCAAGACTGA	TTCATGCCTTCTTTCACTTTGC
RPL13A	CCTGGAGGAGAAGAGGAAAGAGA	TTGAGGACCTCTGTGTATTTGTCAA
**Target genes**		
BNIP3	AGCGCCCGGGATGCA	CCCGTTCCCATTATTGCTGAA
BNIP3L	CTGCGAGGAAAATGAGCAGTCTCT	GCCCCCCATTTTTCCCATTG
CCL2	AGCAGCAAGTGTCCCAAAGAAGCT	CCTTGGCCACAATGGTCTTGAA
MT-CO2	ACCTGCGACTCCTTGACGTT	GGGGGCTTCAATCGGGAGTA
CYC1 (CIII)	GAGCACGACCATCGAAAACG	CGATATGCCAGCTTCCGACT
DNM1L	CGACTCATTAAATCATATTTTCTCATTGTCAG	TGCATTACTGCCTTTGGCACACT
FIS1	CCTGGTGCGGAGCAAGTACAA	TCCTTGCTCCCTTTGGGCAG
FOXJ1	TCGTATGCCACGCTCATCTG	CTTGTAGATGGCCGACAGGG
FUNDC1	GAAACGAGCGAACAAAGCAG	GCAAAAAGCCTCCCACAAAT
GABARAPL1	ATCGGAAAAAGGAAGGAGAAAAGATC	CAGGCACCCTGGCTTTTGG
HK2	GTAAATACAGTGGATCTCAATCTTCGGG	CAAGGATTTGAGATGATTCGCTATTCA
IL-8	TTAGAACTATTAAAACAGCCAAAACTCCACA	CAAGTTTCAACCAGCAAGAAATTACTAA-TATTG
KRT5	GGAGTTGGACCAGTCAACATC	TGGAGTAGTAGCTTCCACTGC
KRT14	TGGATCGCAGTCATCCAGAG	ATCGTGCACATCCATGACCT
MAP1LC3A	CCTGGACAAGACCAAGTTTTTG	GTCTTTCTCCTGCTCGTAGATG
MAP1LC3B	ACCATGCCGTCGGAGAAGAC	TCTCGAATAAGTCGGACATCTTCTACTCT
MFN1	CTGAGGATGATTGTTAGCTCCACG	CAGGCGAGCAAAAGTGGTAGC
MFN2	TGGACCACCAAGGCCAAGGA	TCTCGCTGGCATGCTCCAC
MUC5AC	AGCAGGGTCCTCATGAAGGTGGAT	AATGAGGACCCCAGACTGGCTGAA
NDUFB3 (CI)	TCAGATTGCTGTCAGACATGG	TGGTGTCCCTTCTATCTTCCA
NRF2	CTCACCTGGGAACAGAACAGGAA	ACCCAAGAAATGCAGTCTCGAGC
OPA1	TACCAAAGGCATTTTGTAGATTCTGAGTT	GCATGCGCTGTATACGCCAA
PDK4	CCTGTGAGACTCGCCAACA	TCCACCAAATCCATCAGGCTC
PINK1	GAAAGCCGCAGCTACCAAGA	AGCACATTTGCGGCTACTCG
PPARA	CAGAACAAGGAGGCGGAGGTC	AGGTCCAAGTTTGCGAAGC
PPARGC1B	GGCGCTTTGAAGTGTTTGGTGA	TGATGAAGCCGTACTTCTCGCCT
PPRC1	GCCCTTTGATCTCTGCTTTGGG	AAGTCTTCCCGGTTGGAGTCAAG
PRKN	GGTTTGCCTTCTGCCGGGAATG	CTTTCATCGACTCTGTAGGCCTG
SCGB1A1	TTCAGCGTGTCATCGAAACCC	ACAGTGAGCTTTGGGCTATTTTT
SOD1	GGTCCTCACTTTAATCCTCTAT	CATCTTTGTCAGCAGTCACATT
SOD2	TGGACAAACCTCAGCCCTAACG	TGATGGCTTCCAGCAACTCCC
SQSTM1	GGTGCACCCCAATGTGATCT	CGCAGACGCTACACAAGTCG
TFAM	GAAAGATTCCAAGAAGCTAAGGGTGATT	TCCAGTTTTCCTTTACAGTCTTCAGCTTTT
TP63	CCCGTTTCGTCAGAACACAC	CATAAGTCTCACGGCCCCTC

Abbreviations: *ACTB*: Actin B, *B2M*: Beta-2 Microglobulin, *PPIA*: Peptidylprolyl Isomerase A, *RPL13A*: Ribosomal Protein L13A, *BNIP3*: BCL2 Interacting Protein 3, *BNIP3L*: BCL2 Interacting Protein 3-Like, *CCL2*: C-C Motif Chemokine Ligand 2, *MT-CO2*: Mitochondrially Encoded Cytochrome C Oxidase II, *CYC1*: Cytochrome C1, *DNM1L*: Dynamin 1-Like, *FIS1*: Fission, Mitochondrial 1, *FOXJ1*: Forkheid Box J1, *FUNDC1*: FUN14 Domain Containing 1, *GABARAPL1*: GABA Type A Receptor Associated Protein Like 1, *HK2*: Hexokinase 2, *IL-8*: Interleukin 8, *KRT5*: Keratin 5, *KRT14*: Keratin 14, *MAP1LC3A*: Microtubule-Associated Protein 1 Light Chain 3 Alpha, *MAP1LC3B*: Microtubule-Associated Protein 1 Light Chain 3 Beta, *MFN1*: Mitofusin 1, *MFN2*: Mitofusin 2, *MUC5AC*: Mucin 5AC, Oligomeric Mucus/Gel-Forming, *NDUFB3*: NADH:Ubiquinone Oxidoreductase Subunit B3, *NRF2*: Nuclear Respiratory Factor 2, *OPA1*: OPA1, Mitochondrial Dynamin Like GTPase, *PDK4*: Pyruvate Dehydrogenase Kinase 4, *PINK1*: PTEN

Induced Kinase 1, *PPARA*: Peroxisome Proliferator Activated Receptor Alpha, *PPARGC1B*: Peroxisome Proliferator-Activated Receptor Gamma, Coactivator 1 Beta, *PPRC1*: Peroxisome Proliferator-Activated Receptor Gamma, Coactivator-Related 1, *PRKN:* Parkin RBR E3 Ubiquitin Protein Ligase, *SOD1*: Superoxide Dismutase 1, *SOD2:* Superoxide Dismutase 2, *SCGB1A1*: Secretoglobin Family 1A Member 1, *SQSTM1*: Sequestosome 1, (transcript variant 1/4), *TFAM*: Transcription Factor A, Mitochondrial, *TP63*: Tumor Protein P63.

Table S3: Target-specific antibodies used for immunohistochemistry staining.

Target	Antibody	Company	Product number	Dilution factor
Acetylated Tubulin	Mouse anti-human Acetylated a tubulin	Sigma	Cat# T7451	1:20.000
Clara cell secretory protein-16	Mouse anti-human CC16, clone AY1E6kn	Sanbio	Cat# HM2178	1:200
P63	Rabbit monocloncal P63	Abcam	Cat# ab124762	1:200

Table S4. Antibodies used for western blotting.

Target	RRID	Company	Product number	Dilution factor
**Primary Antibodies**				
BNIP3	AB_2259284	Cell Signaling Technology	Cat# 3769S	1:1000
BNIP3L	AB_2688036	Cell Signaling Technology	Cat# 12396	1:1000
DNM1L	AB_10950498	Cell Signaling Technology	Cat# 8570	1:1000
ESRRA	AB_1523580	Abcam	Cat# ab76228	1:1000
FUNDC1	AB_10609242	Santa Cruz Biotechnology	Cat# sc-133597	1:500
GABARAPL1	AB_2294415	Proteintech Group	Cat# 11010-1-AP	1:1000
HK2	AB_2232946	Cell Signaling Technology	Cat# 2867	1:1000
MAP1LC3B	AB_915950	Cell Signaling Technology	Cat# 2775	1:1000
NRF1	AB_2154534	Abcam	Cat# ab55744	1:1000
OXPHOS	AB_2629281	MitoScience LLC	Cat# MS604	1:1000
PINK1	AB_10127658	Novus Biologicals	Cat# BC100-494	1:2000
PPARGC1A	AB_10697773	Millipore	Cat# 516557	1:1000
PRKN	AB_2159920	Cell Signaling Technology	Cat# 4211	1:1000
SQSTM1	AB_10624872	Cell Signaling Technology	Cat# 5114	1:1000
TOMM20	AB_2716623	Abcam	Cat# Ab186734	1:1000
**Secondary Antibodies**				
Goat Anti-Mouse IgG Antibody	AB_2827937	Vector Laboratories	Cat#BA-9200	1:10000
Goat Anti-Rabbit IgG Antibody	AB_2313606	Vector Laboratories	Cat#BA-1000	1:10000

Abbreviations: BNIP3: BCL2 Interacting Protein 3, BNIP3L: BCL2 Interacting Protein 3-Like, DNM1L: Dynamin 1-Like, ESRRA: Estrogen Related Receptor, Alpha, FUNDC1: FUN14 Domain Containing 1, GABARAPL1: GABA Type A Receptor Associated Protein Like 1, HK2: Hexokinase 2, MAP1LC3B: Microtubule-Associated Protein 1 Light Chain 3 Beta, NRF1: Nuclear Respiratory Factor 1, OXPHOS: oxidative phosphorylation antibody cocktail (containing NDUFB8: NADH:Ubiquinone Oxidoreductase Subunit B8 (CI), SDHB: Succinate Dehydrogenase Complex Iron Sulfur Subunit B (CII), UQCRC2: Ubiquinol-Cytochrome C Reductase Core Protein 2 (CIII), ATP5F1A: ATP Synthase F1 Subunit Alpha (CV)), PINK1: PTEN Induced Kinase 1, PPARGC1A: Peroxisome Proliferator-Activated Receptor Gamma, Coactivator 1 Alpha, PRKN: Parkin RBR E3 Ubiquitin Protein Ligase, SQSTM1: Sequestosome 1, TOMM20: Translocase Of Outer Mitochondrial Membrane 20.

References

1. Pauwels, C.; Klerx, W.N.M.; Pennings, J.L.A.; Boots, A.W.; van Schooten, F.J.; Opperhuizen, A.; Talhout, R. Cigarette Filter Ventilation and Smoking Protocol Influence Aldehyde Smoke Yields. *Chem Res Toxicol* **2018**, *31*, 462-471, doi:10.1021/acs.chemrestox.7b00342.
